# Intraperitoneal drug delivery systems releasing cytostatic agents to target gastro-intestinal peritoneal metastases in laboratory animals: a systematic review

**DOI:** 10.1007/s10585-022-10173-8

**Published:** 2022-06-23

**Authors:** Anne G. W. E. Wintjens, Geert A. Simkens, Peter-Paul K. H. Fransen, Narcis Serafras, Kaatje Lenaerts, Gregor H. L. M. Franssen, Ignace H. J. T. de Hingh, Patricia Y. W. Dankers, Nicole D. Bouvy, Andrea Peeters

**Affiliations:** 1 NUTRIM School of Nutrition and Translational Research in Metabolism, Maastricht University, Maastricht, The Netherlands; 2 Department of Surgery, Maastricht University Medical Centre, PO Box 616, 6200 MD Maastricht, The Netherlands; 3 Department of Surgery, Catharina Hospital Eindhoven, Eindhoven, The Netherlands; 4 UPyTher BV, Eindhoven, The Netherlands; 5 Department of Education, Content & Support, University Library, Maastricht University, Maastricht, The Netherlands; 6 GROW School for Oncology and Developmental Biology, Maastricht University, Maastricht, The Netherlands; 7 Institute for Complex Molecular Systems, Eindhoven University of Technology, Eindhoven, The Netherlands; 8 Department of Biomedical Engineering, Laboratory of Chemical Biology, Eindhoven University of Technology, Eindhoven, The Netherlands; 9 Department of Clinical Epidemiology and Medical Technology Assessment, Maastricht University Medical Centre, Maastricht, The Netherlands

**Keywords:** Systematic review, Drug delivery systems, Intraperitoneal chemotherapy, Peritoneal metastases, Animal experiments

## Abstract

For peritoneal metastases (PM), there are few curative treatment options, and they are only available for a select patient group. Recently, new therapies have been developed to deliver intraperitoneal chemotherapy for a prolonged period, suitable for a larger patient group. These drug delivery systems (DDSs) seem promising in the experimental setting. Many types of DDSs have been explored in a variety of animal models, using different cytostatics. This review aimed to provide an overview of animal studies using DDSs containing cytostatics for the treatment of gastro-intestinal PM and identify the most promising therapeutic combinations. The review was conducted following the Preferred Reporting Items for Systematic Reviews and Meta-Analyses (PRISMA) guidelines and Systematic Review Center for Laboratory Animal Experimentation (SYRCLE) guidelines. The 35 studies included revealed similar results: using a cytostatic-loaded DDS to treat PM resulted in a higher median survival time (MST) and a lower intraperitoneal tumor load compared to no treatment or treatment with a ‘free’ cytostatic or an unloaded DDS. In 65% of the studies, the MST was significantly longer and in 24% the tumor load was significantly lower in the animals treated with cytostatic-loaded DDS. The large variety of experimental setups made it impossible to identify the most promising DDS-cytostatic combination. In most studies, the risk of bias was unclear due to poor reporting. Future studies should focus more on improving the clinical relevance of the experiments, standardizing the experimental study setup, and improving their methodological quality and reporting.

## Introduction

The peritoneal cavity is a common location for metastases from a large variety of malignancies.

Peritoneal metastases (PM) originate most commonly from the primary tumors of gastro-intestinal, reproductive, and genitourinary tracts. Although, they can also be caused by other malignancies such as breast- or lung cancer [1]. The incidence of PM from colorectal origin is estimated to be 10–13% [2, 3]. The incidences of PM from gastric and pancreatic origin are similar, with estimates up to 21% and 9–14% respectively [4–7]. However, it is difficult to detect PM due to their small size and the limited contrast resolution available with routine imaging, so the reported incidence of PM is probably an underestimation [8].

Historically, after being diagnosed with PM, patients faced a poor prognosis with best supportive care as the main treatment option [9]. The introduction of systemic chemotherapy improved their prognosis, but unlike other metastatic sites, PM tend to have a limited response to systemic chemotherapy [10, 11]. The search for local and more effective treatment strategies resulted in the implementation of cytoreductive surgery (CRS) followed by hyperthermic intraperitoneal chemotherapy (HIPEC). Several randomized controlled trials and large cohort series reported improved median survival rates of 21.6 up to 41.7 months among patients with colorectal PM treated with CRS and HIPEC, but the outcome was highly dependent on patient selection [12–14]. Nevertheless, this multimodality treatment continues to be regarded as a viable treatment option in selected, fit colorectal cancer patients with limited PM and no systemic metastases. Unfortunately, due to strict contra-indications, only 10–25% of patients with PM of colorectal origin are eligible for CRS and HIPEC [15, 16]. For PM of non-colorectal gastro-intestinal origins such as gastric or pancreatic adenocarcinoma, CRS and HIPEC are considered experimental because of limited available evidence and poor survival [17, 18]. For those patients not eligible for CRS and HIPEC, pressurized intraperitoneal aerosol chemotherapy (PIPAC) is a new palliative treatment option that is considered safe. Randomized research is needed to confirm its additional value [19–23].

Despite the fact that these recent achievements have improved the prognosis of PM patients, treatment failure often occurs and so the desire for new and improved therapies remains. In the experimental setting, much effort has therefore been devoted to developing a novel 'Drug Delivery System' (DDS). The rationale behind these DDSs is that a higher intraperitoneal chemotherapy concentration can be administered for a prolonged period and with limited systemic side effects, which would make them viable for a wide variety of patients in different stages of the disease. Many types of DDS have already been explored in animal models for PM, e.g. hydrogels, microspheres, nanoparticles, microparticles, and liposomes. These types of DDSs can carry many different cytostatic agents and have been applied in a wide variety of animal models for PM. Such a large diversity of combinations, however, makes it difficult to determine which combination of DDS and cytostatic agent yields the most promising result.

This systematic review aims to provide a comprehensive overview of the current animal studies using a DDS carrying a cytostatic drug for the treatment of PM of gastro-intestinal origin. The goal is to identify the most promising combination of DDS and cytostatic agent in animal models. With this information, recommendations may be defined to further improve research in this field.

## Methods

### Protocol and registration

This systematic review was registered at PROSPERO international prospective register of systematic reviews [registration number: CRD42020207678]. It was conducted and reported in accordance with the Preferred Reporting Items for Systematic Reviews and Meta-Analyses (PRISMA) guidelines and the Systematic Review Center for Laboratory Animal Experimentation (SYRCLE) guidelines.

### Search strategy

PubMed and Embase were systematically searched on 10 September 2020 and on 14 December 2021. Free-text terms, MeSH terms, and Emtree’s regarding ‘peritoneal metastases', ‘drug delivery systems’, and ‘animal’ (the latter by using PubMed and Embase search filters of SYRCLE) were used to search both databases. The full search strategy is available in appendix 1. A professional clinical librarian (GF) was involved to ensure a correct searching strategy.

### Inclusion and exclusion criteria

An article was eligible for inclusion if the following criteria were met: (1) the study described an in vivo experiment in which PM of gastro-intestinal origin was induced via intraperitoneal inoculation with tumor cells (either syngeneic or xenograft), (2) induced PM was treated with any type of an intraperitoneal delivered DDS containing a chemotherapeutic agent currently used in clinical practice to treat all types of PM, as summarized by Valle et al. [24], (3) the experiment was an intervention study with at least two groups (intervention and control group), (4) follow-up of animals after exposure to treatment was at least one week, (5) reported outcomes of the experiment were survival and/or reduction in intraperitoneal tumor load after exposure to therapy. Articles published before the year 2000 were excluded. Only articles written in the English language were included. Human trials, in vitro, and ex vivo experiments were excluded. Conference abstracts and unpublished results were not considered.

### Study selection

All search results were imported in a free web tool designed for systematic reviewers (Rayyan) [25]. All duplicates were removed. Studies were screened in two stages. Two researchers (AW and NS) independently pre-screened the titles and abstracts of all articles before assessing the full-texts of all articles eligible based on the titles and abstracts. The researchers were blinded to each other’s decision when performing the full-text assessment. Disagreement was resolved by initial discussion and, if needed, a senior researcher (GS) was consulted to make a final decision.

### Data extraction

Two researchers (AW and NS) extracted the data of all eligible articles separately using a standardized, pre-piloted datasheet. Data were extracted from text, tables, and/or figures. Disagreement was resolved by initial discussion and, if needed, a senior researcher (GS) was consulted to make a final decision. The following data were extracted: general study characteristics (first author and publication year), animal characteristics (species, strain, and sex of the animals), type of tumor (cell line, number of cells used for inoculation, number of days between inoculation and start treatment), intervention (type of DDS, type and dosage of cytostatic, experiment duration, DDS administration frequency), and outcomes (tumor load quantified as mean intraperitoneal tumor weight, tumor volume, number of tumor nodules, or signal intensity measured by an in-vivo imaging system, and median survival time).

### Study quality assessment

The quality of integrated studies was assessed using the SYRCLE’s risk of bias tool, an adapted version of the Cochrane risk of bias tool specifically developed for animal studies [26]. Selection bias, performance bias, detection bias, attrition bias, and reporting bias were assessed, again by two independent researchers (AW and NS).

### Synthesis of results

Results of the included studies were descriptively summarized. Median survival times were displayed in tables and text as reported by the studies' authors. This could either be defined as a time from inoculation to survival endpoint, or a time from administration of the treatment to survival endpoint. The outcomes of the statistical analyses reported by the authors were used. It was impossible to perform a meta-analysis due to the large heterogeneity in terms of the type of DDS, the choice of cytostatic agent, and the type of tumor cell line.

## Results

### Identification of relevant studies

**Fig. 1:**
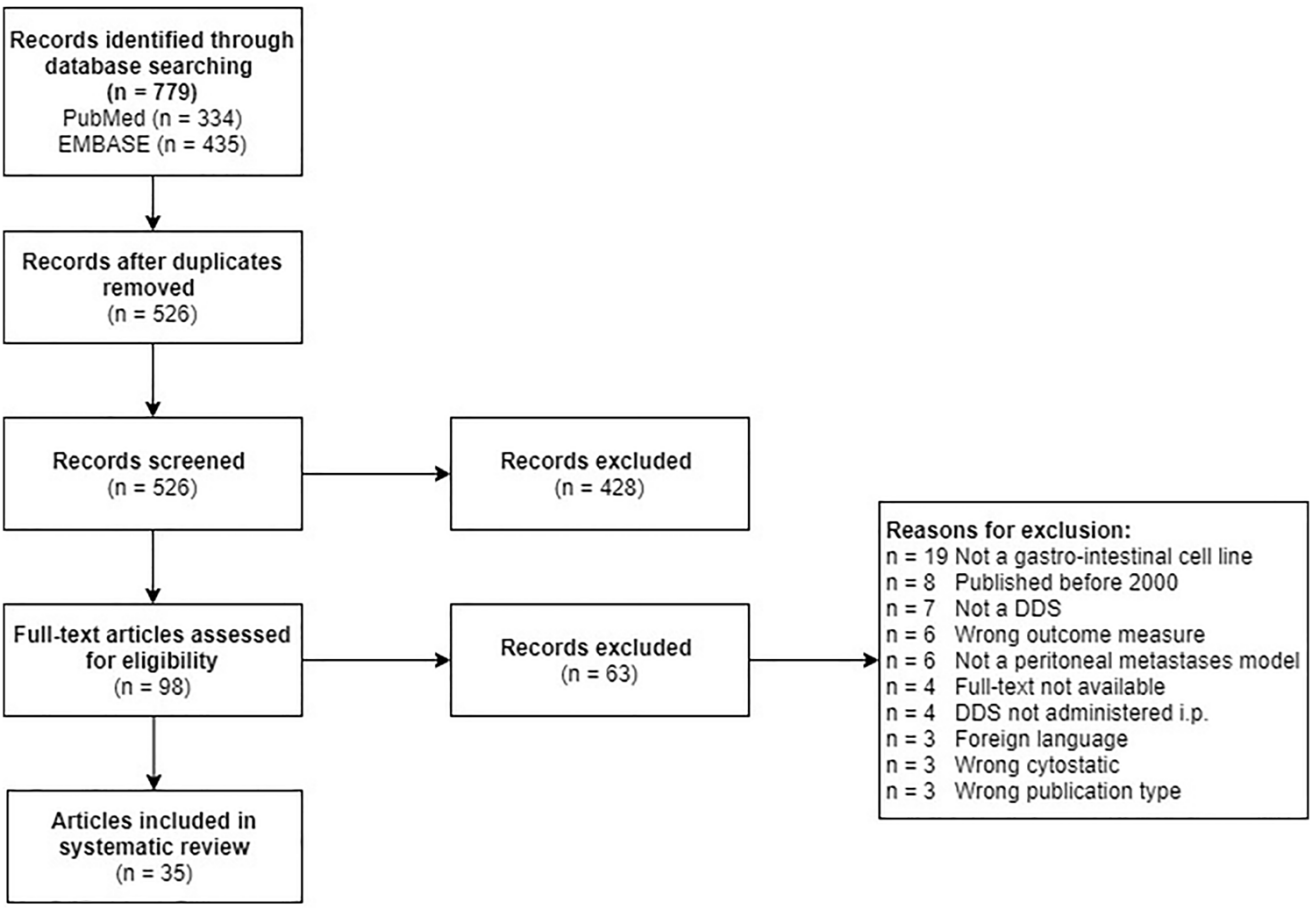
Flow diagram of included studies

After duplicates had been removed, 526 potentially relevant articles were identified. After the abstracts had been read, 428 articles were excluded because they met the predefined exclusion criteria. The remaining 98 articles underwent full-text assessment; 63 papers were yet excluded. A total of 35 articles fulfilled the predefined inclusion criteria. The flow diagram of the included studies is visualized in Fig. 1.

### Characteristics of the included studies

All 35 articles included described experimental studies and were published between 2000 and 2020. Table 1 describes the types of DDSs, cytostatics, and outcome parameters per gastro-intestinal cell line used to induce PM. The study characteristics are displayed in Tables 2, 3, and 4. There is large heterogeneity in terms of the type of DDS and the choice of cytostatic, but there are also similarities between the articles in terms of type of animal model/strain and choice of tumor cell line. Both the differences and the similarities will be discussed in the following sections.

**Table 1:** Overview of type of DDSs, type of cytostatics, and outcomes per gastro-intestinal cell line

	PM of colorectal origin, n = 16 (references in parentheses)	PM of gastric origin, n = 14 (references in parentheses)	PM of pancreatic- or liver origin, n = 7 (references in parentheses)	Total
**Type of DDSs**				
Hydrogel	4 [42, 46–48]	5 [40, 41, 68–70]	0	9
Hydrogel containing micelles	2 [31, 33]	0	0	2
Hydrogel containing microspheres	1 [32]	0	0	1
Hydrogel containing nanoparticles	1 [37]	1 [49]	0	2
Microsphere	3 [43–45]	2 [27, 71]	1 [27]	6
Nanoparticle	1 [72]	2 [39, 73]	1 [74]	4
Polymerosome	1 [28]	2 [28, 75]	0	3
Micelle	2 [38, 76]	0	0	2
Microparticle	0	0	2 [36, 58]	2
Drug eluting beads	1 [34]	0	1 [35]	2
Liposome	0	1 [77]	0	1
Carrier erythrocyte	0	0	1 [78]	1
Micellar nanoparticle formulation	0	1 [30]	0	1
Micelles, nanoparticles, and microparticles	0	0	1 [29]	1
**Type of cytostatics**				
Paclitaxel	3 [28, 38, 48]	7 [28, 30, 39, 49, 70, 73, 75]	4 [29, 36, 58, 74]	14
Cisplatin	1 [45]	4 [27, 68, 69, 77]	1 [27]	6
5-FU	3 [42, 46, 72]	0	1 [78]	4
Doxorubicin	3 [31, 34, 47]	0	1 [35]	4
Docetaxel	1 [44]	2 [40, 41]	0	3
Docetaxel + LL37	1 [37]	0	0	1
Docetaxel + curcuma	1 [43]	0	0	1
Mitoxantrone	1 [34]	0	1 [35]	2
Floxuridine	0	1 [71]	0	1
Irinotecan	0	0	1 [35]	1
*Simultaneously delivered*				
5-FU + cisplatin + paclitaxel	1 [32]	0	0	1
Paclitaxel + 5-FU	1 [76]	0	0	1
5-FU + cisplatin	1 [33]	0	0	1
**Outcome parameters**				
Tumor number/tumor weight/tumor volume	16 [28, 31–34, 37, 38, 42–48, 72, 76]	11 [27, 28, 30, 39–41, 49, 68, 71, 73, 75]	3 [27, 35, 74]	30
Median survival time	10 [31–33, 37, 38, 43–45, 47, 76]	6 [39–41, 69, 73, 77]	6 [27, 29, 36, 58, 74, 78]	21
Survival rate	1 [42]	0	1 [35]	2
Photon counts	0	2 [69, 70]	0	2

*DDS* drug delivery system, *PM*  peritoneal metastases, *5-FU* 5-fluorouracil

**Table 2:** Study characteristics of studies using a PM model of colorectal cancer origin

First author (ref)	Species, strain, sex	Type of tumor cell line, injection location, and number of cells administered to induce PM	Time between tumor inoculation and start therapy (days)	Type and dosage of cytostatic agent administered	Type of DDS administered	Frequency of DDS administration	Total experiment duration starting from tumor inoculation (days)
Bae et al. [46]	Mouse BALB/c Sex not stated	CT-26-Luc IP 1 × 10^5^	1	5-FU 100 mg/kg	Thermo-responsive conjugated linoleic acid-coupled Pluronic F-127 Poloxamer hydrogel (Plu-CLA)	1	10
Chen et al. [47]	Mouse BALB/c Female	CT-26 ± Luc IP 2 × 10^5^	7	Doxorubicin 1 mg/kg	Thermo-sensitive hyaluronic acid-g-chitosan-g-poly(N-isoropylacrylamide) hydrogel	1	21 and until survival endpoint was reached*
Cherukula et al. [38]	Mouse BALB/c Female	CT-26 IP 5 × 10^5^	10	Paclitaxel 10 mg/kg	Lithocholic acid-conjugated disulfide-linked polyethyleneimine micelle	1	25
		HCT-116 IP 6 × 10^7^	7				19
Fan et al. [44]	Mouse BALB/c Male	CT-26 IP 2 × 10^5^	7	Docetaxel 4–8 mg/kg	PLLA-L121-PLLA microsphere	Once a week	Until survival endpoint was reached*
Fan et al. [37]	Mouse BALB/c Sex not stated	HCT-116 IP 5 × 10^6^	10	Docetaxel + LL37 peptide 8–16 mg/kg	Nanoparticle in a thermo-sensitive PLA-L65-PLA hydrogel	Once a week	30 and until survival endpoint was reached*
Fan et al. [43]	Mouse BALB/c Sex not stated	CT-26 IP 2 × 10^5^	7	Docetaxel + Curcumin 8 mg/kg	PLFL nanofibrous microspheres	1	15 and until survival endpoint was reached*
Gong et al. [76]	Mouse BALB/c Female	CT-26 IP 2 × 10^5^	7	Doxorubicin 5 mg/kg	PECE micelles	1	21 and until survival endpoint was reached*
Gong et al. [31]	Mouse BALB/c Both sexes	CT-26 IP 2 × 10^5^	5	Paclitaxel 2–4 mg/kg FU 2–4 mg/kg	PTX-encapsulated PCEC micelles and a FU-loaded thermo-sensitive PCEC hydrogel	1	20 and until survival endpoint was reached*
Gunji et al. [45]	Mouse BALB/c Male	CT-26 IP 1 × 10^6^	1	Cisplatin 10–20 mg/kg	Gelatin microspheres	2 (day 2 and 5)	10 and until survival endpoint was reached*
Keese et al. [34]	Mouse BALB/c Female	EGFP-C-26 IP 1 × 10^6^	7 or 12	Mitoxantrone 20 mg/kg Doxorubicin 25 mg/kg	Polyvinyl-alcohol hydrogel drug eluting beads	1 (day 12) or 3 (day 7, 10, and 12)	15
Luo et al. [32]	Mouse BALB/c Female	CT-26 IP 2 × 10^5^	7	Paclitaxel 5 mg/kg Cisplatin 1 mg/kg 5-FU 20 mg/kg	HA encapsulated PCEC microspheres	2 (once a week)	21 and until survival endpoint was reached*
Simon-Gracia et al. [28]	Mouse BALB/c Sex not stated	CT-26 IP 0.5 × 10^6^ SC 0.5 × 10^6^	4	Paclitaxel 4.5 mg/kg	iRGD POEGMA-PDPA polymerosomes	4 (every other day)	12
Tang et al. [72]	Mouse BALB/c Sex not stated	HCT116 IP 5 × 10^5^	7	5-FU 40 mg/kg	PEG-PLGA nanoparticles	4 (once a week)	28
Wang et al. [42]	Mouse BALB/c Female	CT-26 IP 2 × 10^5^	5	5-FU 25 mg/kg	PECE thermo-sensitive hydrogel	2 (once a week)	20
Xu et al. [48]	Mouse BALB/c Female	CT-26 IP 1 × 10^5^	5	Paclitaxel 30 mg/kg	Thermo-sensitive PECT hydrogel	1	15 or 25
Yun et al. [33]	Mouse BALB/c Female	CT-26 IP 2 × 10^5^	7	5-FU 20 mg/kg Cisplatin 1 mg/kg	Polymeric micelles in a thermo-sensitive chitosan hydrogel	1	21 and until survival endpoint was reached*

5-FU = 5-fluorouracil; DDS = drug delivery system; HA = hyaluronic acid; IP = intraperitoneal; Luc = luciferase transfected; PCEC = poly(ε-caprolactone)-poly(ethylene glycol)-poly(ε-caprolactone); PECT = poly(ε-caprolactone-co-1,4,8-trioxa [4.6]spiro-9-undecanone)-polu(ehyleneglycol)-poly(ε-caprolactone-co-1,4,8-trioxa [4.6]spiro-9-undecanone); PEG-PLGA = poly(ethylene glycol)-poly(lactic acid-co-glycolic acid); PLA = polylactic acid; PLFL = polyactic acid-Pluronic F68-polyactic; PLLA-L121-PLLA = poly(L-actide acid)-Pluronic L121-poly (L-actide acid); PM = peritoneal metastases; POEGMA-PDPA = Poly(oligoethylene glycol methacrylate)-poly(2-diisopropylamino)ethyl methacrylate); PTX = paclitaxel; SC = subcutaneous

*Part of the animals were kept in the experiment for determining tumor load at a certain day, other part was followed to determine median survival time

**Table 3:** Study characteristics of studies using a PM model of gastric cancer origin

First author (ref)	Species, strain, sex	Type of tumor cell line, injection location, and number of cells administered to induce PM	Time between tumor inoculation and start therapy (days)	Type and dose of cytostatic agent administered	Type of DDS administered	Frequency of DDS administration	Total experiment duration starting from tumor inoculation (days)
Bae et al. [40]	Mouse BALB/c Male	TMK1 IP 1 × 10^7^	7	Docetaxel 10 mg/kg	Thermo-responsive Plu-CLA hydrogel	1	28 and until survival endpoint was reached*
Emoto et al. [30]	Mouse BALB/c Female	MKN45P IP 2 × 10^6^ SC 1 × 10^6^	7	Paclitaxel 40 mg/kg	NK105 polymeric micellar nanoparticle formulation	2 (day 7 and 14)	19
Emoto et al. [68]	Mouse BALB/c Female	MKN45P IP 1 × 10^6^	7	Cisplatin 1 mg/kg	In situ cross-linkable hyaluronic acid-based hydrogel	3 (day 7, 14, and 21)	28
Han et al. [41]	Mouse BALB/c Female	44As3Luc IP 1 × 10^6^	3	Docetaxel 2–8 mg/kg	Polyphosphazene thermo-sensitive hydrogel	1	11, 17, or 31
Iinuma et al. [77]	Mouse BALB/cA JcI-nu Female	MKN45P IP 1 × 10^7^	1	Cisplatin 5 mg/kg	Tf-PEG liposome	2 (day 2 and 5)	60
Inoue et al. [71]	Mouse BALB/c Male	MKN45 IP 2 × 10^6^	7	Floxuridine 1 mg/kg	PLGA microspheres	1	28
Kinoshita et al. [39]	Mouse NCr-nu Female	OCUM-2MD3 IP 1 × 10^7^	7	Paclitaxel 30 mg/kg	Nanoparticle albumin-bound	7 (consecutive days)	25 and until survival endpoint was reached*
Qian et al. [49]	Mouse BALB/c Male	MKN45 IP 5 × 10^6^	14	Paclitaxel 8 mg/kg	Hydrogel-encapsulating paclitaxel-loaded RBC membrane nanoparticles	1	22
Simon-Gracia et al. [28]	Mouse Athymic nude Sex not stated	MKN-45P IP 2 × 10^6^	3	Paclitaxel 7 mg/kg	iRGD pH-sensitive POEGMA-PDPA polymerosomes	8 (every other day)	18
Simon-Gracia et al. [75]	Mouse Athymic nude Sex not stated	MKN45-P-Luc IP 1 × 10^6^	8	Paclitaxel 7 mg/kg	pH-sensitive POEGMA-PDPA polymerosomes	7 (every other day)	21
Soma et al. [73]	Mouse BALB/c Female	MKN45P IP 3 × 10^6^	7	Paclitaxel 20 mg/kg	Amphiphilic polymer composed of PMB-30 W	3 (day 7, 14, and 21)	28 and until survival endpoint was reached*
Tamura et al. [27]	Mouse BALB/cA JcI Female	H-145 IP 3 × 10^6^	7	Cisplatin 20–40 mg/kg	Biodegradable microspheres	1	42
Yamashita et al. [69]	Mouse BALB/c Female	MKN45-Luc IP 5 × 10^6^	5	Cisplatin 5–10 mg/kg	Gelatin hydrogel granules	2	26 and until survival endpoint was reached*
Yu et al. [70]	Mouse BALB/c Female	HSC44Luc IP 1 × 10^6^	3	Paclitaxel 15–30 mg/kg	Biodegradable thermo-sensitive hydrogel	1	5 and 25

DDS = drug delivery system; IP = intraperitoneal; Luc = luciferase transfected; PECE = poly(ethylene glycol)-poly(ε-caprolactone)-poly(-ethylene glycol); PEG = poly(ethylene glycol); PLG = poly(D,L-lactide-co-glycolide); PLGA = poly(lactic acid-co-glycolic acid); Plu-CLA = Pluronic F-127 Poloxamer hydrogel conjugated linoleic acid; PM = peritoneal metastases; PMB-30 W = polymer composed of 2-methacryloxyethyl phosphorylcholine and n-butyl methacrylate; POEGMA-PDPA = Poly(oligoethylene glycol methacrylate)-poly(2-diisopropylamino)ethyl methacrylate); RBC = red blood cell; SC = subcutaneous

*Part of the animals were kept in the experiment for determining tumor load at a certain day, other part was kept in the experiment until survival endpoint was reached to determine median survival time

**Table 4:** Study characteristics of studies using a PM model of pancreatic- and liver cancer origin

First author (ref)	Species, strain, sex	Type of tumor cell line, injection location, and number of cells administered to induce PM	Time between tumor inoculation and start therapy (days)	Type and dose of cytostatic agent administered	Type of DDS administered	Frequency of DDS administration	Total experiment duration starting from tumor inoculation (days)
Herrera et al. [74]	Rat Nude Female	Panc-1-CSC IP 2 × 10^6^	14	Paclitaxel	pH responsive expansile nanoparticles	4 (once a week)	50
Lu et al. [58]	Mouse Nu/Nu Female	Hs766T IP 2 × 10^7^	10	Paclitaxel 40 mg/kg	Polymeric tumor-penetrating PLG microparticles	1	Until survival endpoint was reached*
		MiaPaCa2 IP 2 × 10^7^	15				
Tsai et al. [29]	Mouse Nude BALB/c Female	Hs766T IP 20 × 10^6^	10	Paclitaxel 40 mg/kg	Micelles, gelatin nanoparticles, and polymeric microparticles	1	Until survival endpoint was reached*
Tsai et al. [36]	Mouse Athymic Female	Hs766T IP 20 × 10^6^	10	Paclitaxel Max. cum dose 120 mg/kg	PLGA microparticle	1	Until survival endpoint was reached (max 110 days)*
Yagublu et al. [35]	Mouse C57BL/6 Female	Panc02 IP 1 × 10^6^	15	Mitoxantrone (15–40 mg/kg) Doxorubicin (10–40 mg/kg) Irinotecan (20–30 mg/kg)	Polyvinyl-alcohol hydrogel drug eluting beads	1 (day 15) 3 (day 15–18-21)	24
Tamura et al. [27]	Mouse BALB/cA JcI Female	Li-7 Number of cells not stated	8	Cisplatin 30–35 mg/kg	Biodegradable microspheres	1	Until survival endpoint was reached*
Wang et al. [78]	Mouse Kunming Female	H22 2 × 10^6^	7	5-FU 20 mg/kg	Carrier erythrocyte (RBC)	Twice a week	Until survival endpoint was reached*

DDS = drug delivery system; IP = intraperitoneal; PLG = poly(D,L-lactide-co-glycolide); PLGA = poly(lactic acid-co-glycolic acid); PM = peritoneal metastases; RBC = red blood cell

*Animals were kept in the experiment until survival endpoint was reached to determine median survival time

## Animals and induction of PM

Of the 35 studies, 34 used mice as laboratory animals, the other using laboratory rats. Most often the BALB/c mouse was used (29/35). In 24/35 articles only female animals were used, in 5/35 only males were used, in 1 article both sexes were used, and 5 made no mention of the animal's sex.

Most studies described experiments using only one tumor cell line, but two articles described using two types of tumor cell lines, which are considered here as separate experiments (37 experiments in 35 articles).

In sixteen experiments, PM was induced using a colorectal carcinoma cell line, with the syngeneic CT-26 cell line most often used (n = 13). In one experiment, these cells were transfected with the luciferase gene. Other cell lines used were HCT-11 and EGFP-C-26. PM was induced via intraperitoneal injection with cells number varying between 1 × 10^5^ and 6 × 10^7^. Cells were suspended in growth medium or phosphate-buffered saline (PBS) before injection. The time between tumor inoculation and start of therapy (inoculation period) varied between 1 and 10 days.

In fourteen experiments, PM was induced using a gastric cancer cell line, with MKN-45P used most often (n = 8). The number of cells for this cell line varied between 1 × 10^6^ and 1 × 10^7^; the inoculation period was up to 14 days. Other experiments used TMK1, 44As3, OCUM2MD3, H-154, or HSC44 cells, sometimes transfected with the luciferase gene.

In the remaining experiments, five used a cell line derived from pancreatic carcinoma and two used a liver carcinoma cell line. For pancreatic cancer, Hs766T was chosen most often (n = 3). The cell number varied between 1 × 10^6^ and 20 × 10^6^ cells, whereas the inoculation period for the pancreatic cell lines was much longer, at 10 to 15 days, compared to the colorectal- and gastric cell lines.

There were two studies that included two cell lines: Tamura et al. used both gastric- and liver cancer cell lines [27], and Simón-Gracia et al. used both colon- and gastric cancer cell lines [28]. These are considered here as separate studies.

## DDSs

**Fig. 2:**
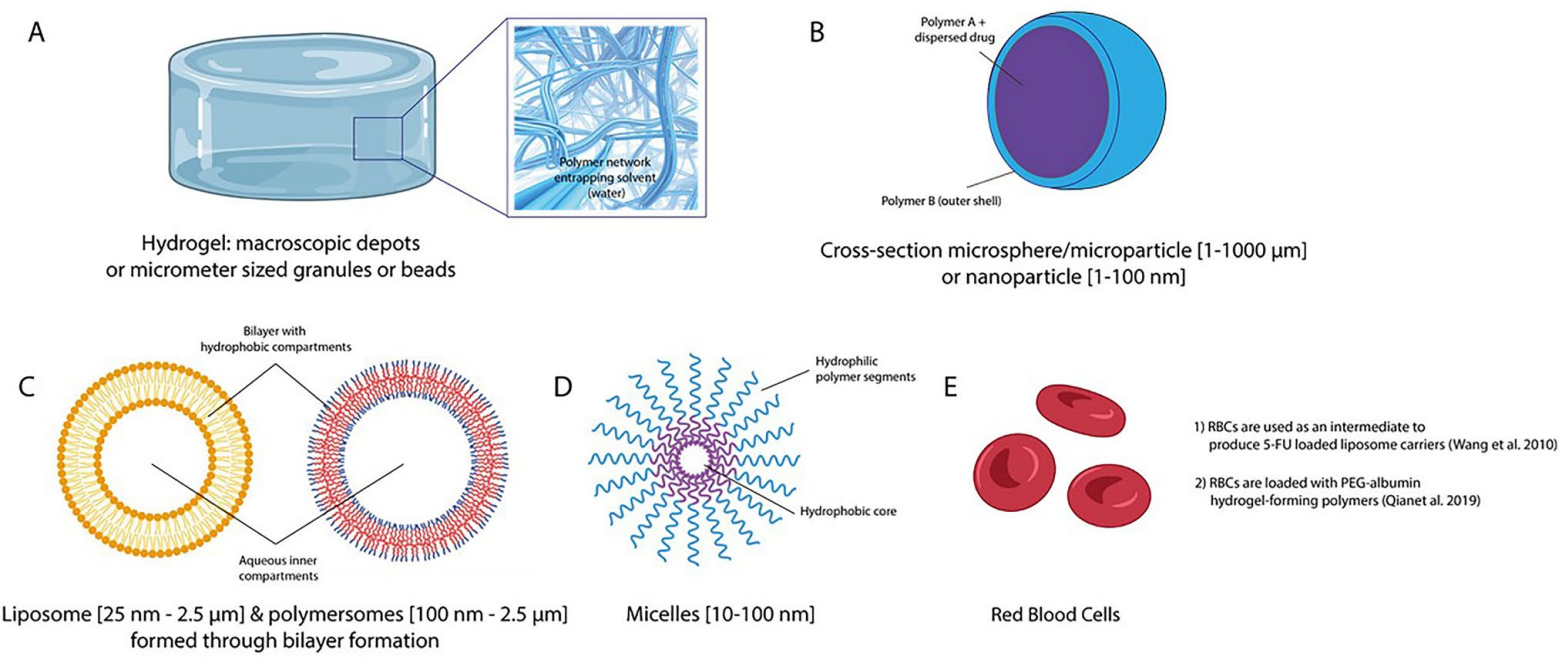
Overview of all included DDSs in this review

The choice of DDS varied between studies. Most often, a variant of a (thermo-responsive) hydrogel system was used (n = 14), sometimes combined with nanoparticles, red blood cell membrane nanoparticles, micelles, or microspheres. Other DDSs used were microspheres (n = 6), nanoparticles (n = 4), polymersomes (n = 3), microparticles (n = 2), micelles (n = 2), drug eluting beads (n = 2), liposomes (n = 1), and carrier erythrocytes (n = 1). Tsai et al. compared the effectiveness of three DDSs: micelles, nanoparticles, and microparticles [29]. Emoto et al. combined micelles and nanoparticles in one formulation [30]. Figure 2 displays all included DDSs.

(A) Hydrogels are polymer networks entrapping a solvent medium, water. Drugs are dissolved in the aqueous medium or are retained in the polymer network, depending on the interaction on the polymer-drug interactions. Usually, hydrogels are used as macroscopic drug depots. Hydrogel granules or beads are micrometer-sized hydrating polymer particles and may be considered hybrid hydrogel-microparticle. (B) Polymer particles are termed micro- or nanoparticles depending on their size or are termed spheres as a result of their shape. Retention of the drug inside the particle is influenced by the polymer-drug interaction and the presence/absence of an outer shell. (C) Liposomes and polymersomes are assembled bilayer systems with aqueous and hydrophobic compartments that enable the retention of different types of drugs. (D) Micelles are polymer nanoparticles composed of block copolymers with hydrophobic and hydrophilic segments that steer assembly. Hydrophobic drugs such as paclitaxel and docetaxel are used as micellular formulations (Taxol or Taxotere). (E) Red blood cells are used to produce liposome carriers (or are combined with hydrogel-forming polymers).

## Cytostatics

There was a great variety in the choice of cytostatics. Paclitaxel was most often used (n = 14), followed by cisplatin (n = 6), 5-FU (n = 4), doxorubicin (n = 4), docetaxel (n = 5), mitoxantrone (n = 2), floxuridine (n = 1), and irinotecan (n = 1). In some studies, a combination of cytostatics was delivered from the DDS simultaneously: paclitaxel – 5-FU, cisplatin – paclitaxel – 5-FU, cisplatin – 5-FU [31–33]. Both Yagublu et al. and Keese et al. compared experimental groups in which different types of cytostatics were administered via drug eluting beads: doxorubicin – mitoxantrone – irinotecan and doxorubicin – mitoxantrone were used, respectively [34, 35].

## Outcome measures

This systematic review focusses on two outcome measures: survival and reduction of intraperitoneal tumor load. The majority of the studies included reported reduction in intraperitoneal tumor load as an outcome (n = 30), and more than half reported survival as outcome (n = 21).

### Risk of bias within studies

The risk of bias was assessed using SYRCLE’s risk of bias tool by applying 10 signaling questions. In general, the reporting of the methodology used was poor, which makes it difficult to assess the risk of bias.

For instance, none of the articles gave any information about the following items: whether the allocation sequence had been adequately generated and applied, whether the allocation to the different experimental groups had been adequately concealed, whether the researchers had randomly placed cages or animals within the room/facility, whether the caregivers and/or researchers had been blinded as to which intervention each animal had received, whether the animals had been randomly selected for outcome assessment, and whether the outcome assessor had been blinded. Thus, the risk of bias for these items is unclear. Only one article gave information about addressing incomplete outcome data; it described how the authors had dealt with missing data [36]. In all other articles, no description was given as to whether all animals had been included in the analysis.

**Fig. 3:**
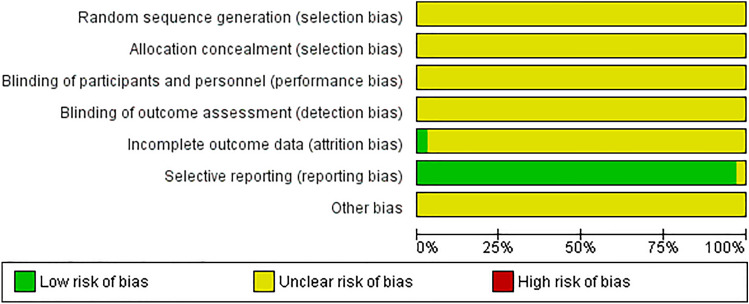
Risk of bias graph presented as percentage of all included studies

For some signaling questions, however, the risk of bias was low. For example, 26 articles gave adequate information about group similarity at baseline (sex, age, and weight of the animals). Another well-described signaling question was whether the reports of the study were free of selective outcome reporting. In 28 papers, the expected outcomes as described in the methods section were also described and analyzed in the result section. Figure 3 displays the risk of bias graph presented as a percentage of all included studies.

### Study outcomes: survival

The first outcome parameter of interest here was survival, which was most often expressed as median survival time (MST). The results regarding this outcome are described in Tables 5, 6, 7, and 8. This outcome parameter is first explained per cell line and thereafter in light of the two most frequently used DDSs (hydrogel and microsphere).

**Table 5:** Study outcomes of studies using a PM model of colorectal cancer origin

First author (ref)	Experimental groups compared (n)	Results – Survival Median survival time (days) ^#^	Results – Tumor load Mean total intraperitoneal tumor weight ± SD (g)	Results – Tumor load Mean tumor volume ± SD (cm^3^)	Results – tumor load Mean number of tumor nodules ± SD	Results – tumor load Signal intensity measured by in-vivo imaging system
Bae et al. [46]	A. Control (n = not reported) B. 5-FU 100 mg/kg i.v. (n = not reported) C. Free 5-FU 100 mg/kg i.p. (n = not reported) D. 5-FU 100 mg/kg i.p. + Plu-CLA 20.8 mM (n = not reported)	n.a	Significant inhibition of tumor growth (p < 0.05) (group comparison not stated)	–	–	–
Chen et al. [47]	A. Control (n = 8) B. Blanc hydrogel (n = 8) C. Free DOX 1 mg/kg (n = 8) D. Hydrogel-DOX 1 mg/kg (n = 8)	A. 18 B. 19 C. 21 D. 29 (N.S.)	A. 2.50 ± 0.12 B. 2.60 ± 0.08 C. 1.13 ± 0.09 D. 0.30 ± 0.03 (vs. A-C p < 0.05)	A. 2.16 ± 0.16 B. 2.70 ± 0.10 C. 1.46 ± 0.12 D. 0.46 ± 0.08 (vs. A-C p < 0.05)	–	Day 7 + 14: A. 38 + 700 B. 34 + 800 C. 1.2 + 1.5 D. 0.5 + 0.5
Cherukula et al. [38]	CT-26 cell line: A. Control (n = 6) B. Blanc micelle (40 mg/kg) (n = 6) C. Free PTX 10 mg/kg (n = 6) D. Micelle-PTX 10 mg/kg (n = 6) HCT-116 cell line: A. Control (n = 4) B. Blanc micelle (40 mg/kg) (n = 4) C. Free PTX 10 mg/kg i.p. (n = 4) D. Micelle-PTX 10 mg/kg (n = 4)	CT-26 cell line: A. 15 B. 18 C. 21 D. 27 (NS) HCT-116 cell line: n.a	CT-26 cell line: A. 6.15 ± 0.4 B. 6.8 ± 0.9 C. 4.7 ± 0.63 D. 1.7 ± 0.7 (vs. B-C p < 0.01, vs. A p < 0.001) HCT-116 cell line: A. 1.35 ± 0.12 B. 1.22 ± 0.1 C. 0.98 ± 0.09 D. 0.32 ± 0.04 (vs. B-C p < 0.01, vs. A p < 0.001)	–	CT-26 cell line: A. 124 ± 12 B. 119 ± 14 C. 98 ± 11 D. 34 ± 8 (vs. C p < 0.01, vs. A-B p < 0.001) HCT-116 cell line: A. 34 ± 6 B. 31 ± 7 C. 24 ± 3 D. 8 ± 2 (vs. A-C p < 0.001)	–
Fan et al. [44]	A. Control (n = 8) B. Blanc microsphere (n = 8) C. Free DOC 4 mg/kg (n = 8) D. Microsphere-DOC 8 mg/kg (n = 8)	A. 23 B. 25 C. 29 D. 33 (vs. A-C p < 0.05)	–	–	*A. 160* ± *30* *B. 148* ± *23* *C. 80* ± *25* *D. 45* ± *5 (vs. A- C p* < *0.05)*	–
Fan et al. [37]	A. Control (n = 12) B. Blanc nanoparticle-hydrogel (n = 12) C. Free DOC 8 mg/kg (n = 12) D. Nanoparticle-hydrogel-DOC 16 mg/kg (n = 12) E. Free DOC + LL37 8 mg/kg (n = 12) F. Nanoparticle-hydrogel DOC + LL37 16 mg/kg (n = 12)	A. 29 B. 35 C. 45 D. 48 E. 49 F. 60 (vs. A-E p < 0.01)	A. 3.07 ± 0.39 B. 3.13 ± 0.3 C. 1.88 ± 0.16 D. 1.66 ± 0.16 E. 1.11 ± 0.10 F. 0.61 ± 0.19 (vs. A-E p < 0.01)	–	A. 73.62 ± 8.68 B. 75.13 ± 4.64 C. 48.04 ± 7.18 D. 42.03 ± 6.36 E. 26.62 ± 4.72 F. 18.21 ± 1.92 (vs A-E p < 0.01)	–
Fan et al. [43]	A. Control (n = 12) B. Blanc microsphere (n = 12) C. Free DOC 8 mg/kg (n = 12) D. Microsphere-DOC (n = 12) E. Free DOC: curcumin (1:1) 8 mg/kg (n = 12) F. Microsphere-DOC + curcumin 8 mg/kg (n = 12)	A. 18 B. 20 C. 29 D. 39 E. 42 F. 48 (vs. A-E p < 0.05)	*A. 3.6* ± *0.5* *B. 3.7* ± *0.6* *C. 2.2* ± *0.3* *D. 1.7* ± *0.35* *E. 1.05* ± *0.3* *F. 0.6* ± *0.3 (vs. A-E p* < *0.05)*	–	*A. 158* ± *30* *B. 143* ± *15* *C. 83* ± *9* *D. 66* ± *20* *E. 58* ± *10* *F. 32* ± *6 (vs. A-E p* < *0.05)*	–
Gong et al. [76]	A. Control (n = 20) B. Blanc micelle (n = 20) C. Free DOX 5 mg/kg (n = 20) D. Micelle-DOX 5 mg/kg (n = 20)	A. 24 B. 23 C. 28 D. 33 (NS)	A. 2.18 ± 0.18 B. 2.14 ± 0.22 C. 0.52 ± 0.15 D. 0.24 ± 0.12 (vs. A-C p < 0.001)	–	A. 50.90 ± 7.71 B. 51.50 ± 6.87 C. 14.10 ± 2.92 D. 6.40 ± 3.78 (vs. A-C p < 0.001)	–
Gong et al. [31]	A. Control (n = 12) B. Blanc micelle-hydrogel (n = 12) C. Free FU 4 mg/kg (n = 12) D. Free PTX 4 mg/kg (n = 12) E. Free PTX 2 mg/kg + FU 2 mg/kg (n = 12) F. Micelle-PTX-hydrogel-FU (n = 12)	A. 23 B. 24 C. 30 D. 32 E. 35 F. 42 (NS)	*A. 3.2* ± *0.6* *B. 3.3* ± *0.5* *C. 1.2* ± *0.25* *D. 1.35* ± *0.2* *E. 0.95* ± *0.25* *F. 0.4* ± *0.35 (vs. A-E p* < *0.001)*	–	*A. 122* ± *52* *B. 126* ± *42* *C. 62* ± *17* *D. 64* ± *18* *E. 36* ± *16* *F. 16* ± *13* (vs. A-C p < 0.001)	–
Gunji et al. [45]	Outcome tumor load: A. Control (n = 5) B. Blanc microsphere (n = 5) C. Free CDDP 10 mg/kg (n = 5) D. Microsphere-CDDP 10 mg/kg (n = 5) Outcome survival: A. Control (n = 6) B. Blanc microsphere (n = 6) C. Free CDDP 20 mg/kg (n = 6) D. Microsphere-CDDP 20 mg/kg (n = 6)	A. 18 B. 25 C. 40 ± 23 D. 74 ± 23 (vs. C p < 0.05)	A. 0.869 ± 0.452 B. 1.070 ± 0.635 C. 0.151 ± 0.066 D. 0.108 ± 0.001 (vs. A p < 0.001)	–	–	–
Keese et al. [34]	Doxorubicin: A. Blanc drug eluting beads (n = 8) B. Free DOX 1 × 10 mg/kg (n = 8) C. Drug eluting beads-DOX 1 × 25 mg/kg (n = 8) D. Free DOX 3 × 10 mg/kg (n = 8) E. Drug loaded beads-DOX 3 × 25 mg/kg (n = 8) F. Free DOX 1 × 100 mg/kg (n = 8) Mitoxantrone: A. Blanc drug eluting beads (n = 8) B. Free MIT 1 × 10 mg/kg (n = 8) C. Drug eluting beads-MIT 1 × 20 mg/kg (n = 8) D. Free MIT 3 × 10 mg/kg (n = 8) E. Drug loaded beads-MIT 3 × 20 mg/kg (n = 8) F. Free MIT 1 × 100 mg/kg (n = 8)	n.a	–	*Doxorubicin:* *A. 13 *[12–27] *B. 2 [1.5–2.5]* *C. 14 *[12–17] *D. n.a* *E. 1.5* *F. 0.2 [0.15–0.23]* *Mitoxantrone:* *A. 48 [37–87]* *B. 6 [5.5–17]* *C. 23 [20.5–37]* *D. 0* *E. 0 [0–0.2] (vs. A p* < *0.05)* *F. 0.3 *[1–8]* (vs. A p* < *0.05)*	–	–
Luo et al. [32]	A. Control (n = 10) B. Blanc hydrogel (n = 10) C. Free 5-FU 20 mg/kg, free PTX 5 mg/kg, free DDP 1 mg/kg (n = 10) D. Drug loaded hydrogel (n = 10)	A. 27 B. 26 C. 32 D. 36 (vs. A-C p < 0.05)	–	–	A. 88 ± 5.86 B.76 ± 5.86 C.29 ± 4.04 D. 14 ± 2.08 (vs. A-C p < 0.05)	–
Simon-Gracia et al. [28]	A. Control (n = 4) B. Blanc polymerosome (n = 4) C. Nanoparticle-albumin PTX 4.5 mg/kg cum dose (n = 4) D. Polymerosome-PTX 4.5 mg/kg cum dose (n = 4) E. Polymerosome-PTX-peptide 4.5 mg/kg cum dose (n = 4)	n.a	*A. 1.5* ± *0.2* *B. 1.6* ± *0.1* *C. 1.7* ± *0.1* *D. 0.8* ± *0.05 (vs. A-C p* < *0.01)* *E. 0.5* ± *0.05 (vs. A-D p* < *0.001)*	–	–	–
Tang et al. [72]	A. Control (n = 6) B. Free 5-FU 40 mg/kg (n = 6) C. Nanoparticle-5-FU 40 mg/kg (n = 6)	n.a	–	–	A. 53.5 ± 9.4 B. 37.7 ± 6.3 C. 28.7 ± 4.2	–
Wang et al. [42]	A. Control (n = 10) B. Blanc hydrogel (n = 10) C. Free 5-FU 25 mg/kg (n = 10) D. Hydrogel-5-FU 25 mg/kg (n = 10)	Survival rate (%) A. 62.5 B. 75 C. 62.5 D. 100 (vs. A-C p < 0.05)	–	–	A. 20.2 ± 10.08 B. 23.67 ± 6.98 C. 11.6 ± 3.8 D. 5.3 ± 4.04 (vs. A-C p < 0.05)	–
Xu et al. [48]	A. Control (n = 8) B. Blanc hydrogel (n = 8) C. Free Taxol (n = 8) D. Hydrogel-PTX (n = 8)	n.a	A. 1.22 ± 0.83 B. 1.24 C. 0.76 ± 0.12 D. 0.55 ± 0.14 (vs. A p < 0.01, vs. C p < 0.05)	–	–	–
Yun et al. [33]	A. Control (n = 12) B. Blanc micelle-hydrogel (n = 12) C. Micelle-5-FU 20 mg/kg (n = 12) D. Hydrogel-CDDP 1 mg/kg (n = 12) E. Free 5-FU 20 mg/kg and free CDDP 1 mg/kg (n = 12) F. Hydrogel-CDDP + micelle-5-FU (n = 12)	A. 25 B. 26 C. 31 D. 33 E. 35 F. 43 (NS)	A. 2.31 ± 0.38 B. 2.26 ± 0.28 C. 0.99 ± 0.17 D. 0.9 8 ± 0.13 E. 0.79 ± 0.13 F. 0.49 ± 0.11 (vs. A-D p < 0.001, vs. E p < 0.05)	–	A. 53.83 ± 9.99 ( B. 52.67 ± 6.12 C. 22.5 ± 4.23 D. 23.22 ± 3.56 E. 18.16 ± 3.06 F. 10.33 ± 2.66 (vs. A-D p < 0.001, vs. E p < 0.05)	–

*All values in italics are values derived from figures and* *not exact numbers*

5-FU = 5-fluorouracil; DOX = doxorubicin; CDDP = cisplatin; DOC = docetaxel; MIT = mitoxantrone; NS = not significant; PEG = poly(ethylene glycol); PM = peritoneal metastases; PTX = paclitaxel; SD = standard deviation

^#^ Median survival times as reported by the studies' authors

**Table 6:** Study outcomes of studies using a PM model of gastric cancer origin

First author (ref)	Experimental groups compared (n)	Results – Survival Median survival time (days) ^#^	Results – Tumor load Mean total intraperitoneal tumor weight ± SD (g)	Results – Tumor load Mean tumor volume ± SD (cm^3^)	Results – tumor load Mean number of tumor nodules ± SD	Results – tumor load Signal intensity measured by an in-vivo imaging system Photon counts ± SD
Bae et al. [40]	A. Control (n = 5) B. Free DOC 10 mg/kg (n = 5) C. Hydrogel-DOC 10 mg/kg (n = 5)	A. 28 B. 31 C. 44 (vs. A-B p < 0.05)	–	A. 41.8 ± 6.47 B. 26.8 ± 5.99 C. 18.6 ± 4.67 (vs. A-B p < 0.05)	–	–
Emoto et al. [30]	A. Control (n = 10) B. Free PTX-Cre 40 mg/kg (n = 7) C. Micelle-nanoparticle-PTX 40 mg/kg (n = 7)	n.a	*Median (IQR) (mg)* *A. 250 (160–295)* *B. 55 (45–95)* *C. 20 *[18–23]* (vs. A-B p* < *0.01)*	–	–	–
Emoto et al. [68]	A. Control (n = 6) B. Blanc hydrogel (n = 6) C. Free CDDP 1 mg/kg (n = 6) D. Hydrogel-CDDP 1 mg/kg (n = 6)	n.a	*A. 0.44* ± *0.42* *B. 0.26* ± *0.11* *C. 0.30* ± *0.13* *D. 0.13* ± *0.09 (vs. A p* < *0.05)*	–	–	–
Han et al. [41]	A. Control (n = 10) B. Blanc hydrogel (n = 10) C. Free DOC i.v. 8 mg/kg (n = 10) D. Free DOC i.p. 8 mg/kg (n = 10) E. Hydrogel-DOC 2 mg/kg (n = 10) F. Hydrogel-DOC 8 mg/kg (n = 10)	A. 9.5 B. 17 C. 15 D. 42 E. 27.5 (vs. A-C p < 0.001) F. 102 (vs. A-C p < 0.001, vs. D p = 0.0068)		Day 8; day 14; day 28 A. 0.72; 0.96 B. 0.74; 1.71; 3.65 C. 0.81; 1.93 D. 0.10; 0.55; 2.94 E. 0.24; 0.47; 2.10 F. 0.0081; 0.022; 0.21 (NS)		…
Iinuma et al. [77]	A. Control (n = 10) B. Bare liposome 5 mg/kg (n = 10) C. PEG-CDDP-liposome 5 mg/kg (n = 10) D. Free CDDP 5 mg/kg (n = 10) E. Tf-PEG-liposome 5 mg/kg (n = 10)	*A. 17* *B. 23* *C. 24 (vs. A p* < *0.05)* *D. 25* *E. 40 (vs. A-C p* < *0.05)*	n.a	n.a	n.a	n.a
Inoue et al. [71]	A. Control (n = 6) B. Blanc microsphere (n = 6) C. Free FUDR bolus MTD (n = 6) D. Free FUDR bolus (n = 6) E. Microsphere-FUDR MTD (n = 6)	n.a	*Corrected for control group:* *B. 98* ± *23* *C. 45* ± *5 (vs. D p* < *0.05)* *D. 99* ± *38* *E. 10* ± *10*	–	–	–
Kinoshita et al. [39]	A. Control (n = 5) B. Free PTX 13.4 mg/kg (n = 5) C. Nanoparticle-albumin bound PTX i.v. 30 mg/kg (n = 5) D. Nanoparticle-albumin bound PTX i.p. 30 mg/kg (n = 5)	A. 25 B. 96 C. 126 (vs. B p < 0.05) D. 122 (vs. B p < 0.05)	*A. 1.25* ± *0.25* *B. 0.55* ± *0.10* *C. 0.62* ± *0.10* *D. 0.48* ± *0.14 (NS)*			
Qian et al. [49]	A. Control (n = 5) B. Free PTX 8 mg/kg (n = 5) C. Nanoparticle-PTX 8 mg/kg (n = 5) D. Hydrogel-nanoparticle-PTX 8 mg/kg (n = 5)	n.a	*A. 0.77* ± *0.54* B. 0.63 ± 0.39 C. 0.52 ± 0.25 D. 0.33 ± 0.22 (vs B and C p < 0.05)	–	*A. 98* ± *19* B. 34.25 ± 11.67 C. 29.2 ± 4.87 D. 19.0 ± 8.0 (vs B and C p < 0.05)	–
Simon-Gracia et al. [28]	A. Control (n = 8) B. Polymerosome-PTX 7 mg/kg cum dose (n = 8) C. Nanoparticle-albumin PTX 7 mg/kg cum dose (n = 8) D. Polymerosome-PTX-peptide 7 mg/kg cum dose (n = 8)	n.a	*A. 1.12* ± *0.14* *B. 0.67* ± *0.11 (vs. A p* < *0.05)* *C. 0.77* ± *0.04 (vs. A p* < *0.05)* *D. 0.49* ± *0.06 (vs. C p* < *0.05; vs. A p* < *0.001)*	–	*A. 68* ± *4* *B. 33* ± *3* *C. 42* ± *9* *D. 18* ± *3 (vs. C p* < *0.001; vs. B p* < *0.01)*	–
Simon-Gracia et al. [75]	A. Control (n = 5) B. Free PTX 7 mg/kg cum dose (n = 5) C. Nanoparticle-albumin-PTX 7 mg/kg cum dose (n = 5) D. Polymerosome-PTX 7 mg/kg cum dose (n = 5)	n.a	–	–	A. 85 ± 11 B. 21.5 ± 4.5 (vs. A p < 0.001) C. 21 ± 4 (vs. A p < 0.001) D. 9.5 ± 2.5 (vs. A p < 0.001)	–
Soma et al. [73]	A. Control (n = 18) B. Cremophor (n = 18) C. Polymer (n = 18) D. Free PTX 20 mg/kg (n = 18) E. Polymer-PTX 20 mg/kg (n = 18)	A. 35 B. 35 C. 35 D. 41.8 E. 51.8 (vs. A-D p < 0.05)	*A. 0.84* ± *0.37* *B. 0.80* ± *0.35* *C. 0.88* ± *0.50* D. 0.22 ± 0.14 (vs. A p < 0.05) E. 0.06 ± 0.05 (vs. A and D p < 0.05)	–	*A. 103* ± *20* *B. 110* ± *25* *C. 108* ± *23* D. 35.5 ± 12.5 (vs. A p < 0.05) E. 9.6 ± 8.3 (vs. A and D p < 0.05)	–
Tamura et al. [27]	A. Control (n = 9) B. Free CDDP 8 mg/kg (n = 9) C. Free CDDP 10 mg/kg (n = 9) D. Microsphere-CDDP 20 mg/kg (n = 9) E. Microsphere-CDDP 35 mg/kg (n = 9) F. Microsphere-CDDP 40 mg/kg (n = 9)	n.a	A. 0.75 ± 0.27 B. 0.23 ± 0.10 C. 0.16 ± 0.07 D. 0.13 ± 0.04 (vs. A p < 0.05) E. 0.13 ± 0.06 (NS) F. 0.07 ± 0.02 (vs. A p < 0.05)	–	–	–
Yamashita et al. [69]	A. Control (n = 12) B. Blanc hydrogel granule (n = 12) C. Free CDDP 1 mg/kg (n = 12) D. Free CDDP 2 mg/kg (n = 12) E. Free CDDP 3 mg/kg (n = 12) F. Free CDDP 5 mg/kg (n = 12) G. Hydrogel granule-CDDP 5 mg/kg (n = 12) H. Hydrogel granule-CDDP 10 mg/kg (n = 12)	A. 39 B. 34 C. 34 D. 41 E. 14 F. 10 G. 51 (vs. A p = 0.0012) H. 51 (vs. A p = 0.0012)	–	–	–	A. 61 E + 04 ± 15 B. 48 E + 04 ± 10 G. 21 E + 04 ± 3 (vs A p < 0.05) H. 23 E + 04 ± 2 (vs A p < 0.05)
Yu et al. [70]	A. Control (n = 5) B. Blanc hydrogel (n = 5) C. Free PTX 30 mg/kg (n = 3) D. Hydrogel-PTX 15 mg/kg (n = 5)	n.a	–	–	–	*A. 7.5 E* + *06* *B. 7.0 E* + *06* *C. 0.5 E* + *0 (vs A p* < *0.05)* *D. 0.5 E* + *0 (vs A p* < *0.05)*

All values in italics are values derived from figures and not exact numbers

CDDP = cisplatin; Cre = cremophor; DOC = docetaxel; FUDR = floxuridine; IQR = interquartile range; NS = not significant; PM = peritoneal metastases; PTX = paclitaxel; SD = standard deviation

^#^ Median survival times as reported by the studies' authors

**Table 7:** Study outcomes of studies using a PM model of pancreatic- and liver cancer origin

Fist author (ref)	Experimental groups compared (n)	Results – Survival Median survival time (days) ^#^	Results – Tumor load Mean total intraperitoneal tumor weight ± SD (g)	Results – Tumor load Mean tumor volume ± SD (cm^3^)	Results – tumor load Mean number of tumor nodules ± SD
Pancreas					
Herrera et al. [74]	A. Control (n = 8) B. Blanc nanoparticle (n = 9) C. Free PTX 10 mg/kg (n = 9) D. Nanoparticle-PTX 10 mg/kg (n = 9)	A. 26 B. 29 C. 44 D. not reached (vs. A-B p < 0.05)	–	–	Mean tumor burden ± SD A. not available due to early death *B. 4* *C. 3* ± *2.5* *D. 2.3* ± *1 (NS)*
Lu et al. [58]	Early stage Hs766T cell line: A. Control (n = 6) B. Free PTX 40 mg/kg 1x (n = 12) C. Microparticle-PTX 120 mg/kg 1x (n = 12) Late stage Hs766T cell line: D. Control (n = 6) E. Free PTX 40 mg/kg 1x (n = 9) F. Microparticle-PTX 120 mg/kg 1x (n = 9) Early stage MiaPaCa-2 cell line: G. Control (n = 6) H. Free PTX 40 mg/kg 1x (n = 7) I. Microparticle-PTX 120 mg/kg 1x (n = 7)	Early stage Hs766T cell line: A. 15 B. 30 (vs. A p < 0.01) C. 41 (vs. A p < 0.01, vs. B p < 0.05) Late stage Hs766T cell line: D. 5 E. 8 F. 14 (vs. D-E p < 0.05) Early stage MiaPaCa-2 cell line: G. 21 H. 42 (vs. G p < 0.01) I. 52(vs. G p < 0.01, vs. H p < 0.05)	n.a	n.a	n.a
Tsai et al. [29]	A. Control (n = 12) B. Free PTX 40 mg/kg (n = 15) C. Nanoparticle-PTX 40 mg/kg (n = 7) D. Microparticle-PTX 40 mg/kg (n = 8)	A. 22 B. 31 C. 34 D. 46 (vs. B-C p < 0.01)	n.a	n.a	n.a
Tsai et al. [36]	A. Control (n = 6) B. Blanc microparticle (n = 6) C. Free PTX 40 mg/kg 1x (n = 16) D. Microparticle small fast release 40 mg/kg PTX (n = 7) E. Microparticle small slow release 80 mg/kg PTX (n = 8) F. Microparticle small fast release 40 mg/kg PTX + small slow release 80 mg/kg PTX (n = 8) G. Microparticle small fast release 60 mg/kg PTX + small slow release 60 mg/kg PTX (n = 8) H. Microparticle small fast release 60 mg/kg + large medium release 60 mg/kg (n = 8) I. Microparticle small slow release 40 mg/kg + large medium release 40 mg/kg + small slow release 40 mg/kg (n = 8) J. Free PTX 40 mg/kg 3x (n = 7) K. Microparticle small fast release 40 mg/kg + small slow release 80 mg/kg 2x (n = 8)	A. 14 B. 15 C. 27 D. 36 E. 21 F. 41 (vs. C p < 0.05) G. 47 (vs. C p < 0.05) H. 42 I. 36 J. 33 K. 55 (vs. C p < 0.05)	n.a	n.a	n.a
Yagublu et al. [35]	Doxorubicin: A. Control (n = 8) B. Free DOX 1 × 25 mg/kg (n = 8) C. Drug eluting beads-DOX 1 × 40 mg/kg (n = 8) D. Free DOX 3 × 10 mg/kg (n = 8) E. Drug eluting beads-DOX 3 × 20 mg/kg (n = 8) Mitoxantrone: F. Control (n = 8) G. Free MIT 1 × 30 mg/kg (n = 8) H. Drug eluting beads-MIT 1 × 40 mg/kg (n = 8) I. Free MIT 3 × 15 mg/kg (n = 8) J. Drug eluting beads-MIT 3 × 20 mg/kg (n = 8) Irinotecan: K. Control (n = 8) L. Free IRI 1 × 40 mg/kg (n = 8) M. Drug eluting beads-IRI 1 × 60 mg/kg (n = 8) N. Free IRI 3 × 20 mg/kg (n = 8) O. Drug eluting beads-IRI 3 × 30 mg/kg (n = 8)	*Doxorubicin:* *A. not reached* *B. 22* *C. not reached* *D. 21* *E. 22* *Mitoxantrone:* *F. not reached* *G. not reached* *H. not reached* *I. 21* *J. not reached* *Irinotecan:* *K. not reached* *L. not reached* *M. not reached* *N. not reached* *O. not reached*	–	*Doxorubicin:* *A. 0.225* ± *0.06* *B. 0.03* ± *0.002 (vs. A p* < *0.001)* *C. 0.025* ± *0.001 (vs. A p* < *0.001)* *Mitoxantrone:* *F. 0.33* ± *0.055* *G. 0.04* ± *0.004 (vs. F p* < *0.001)* *H. 0.038* ± *0.001 (vs. F p* < *0.001)* *Irinotecan:* *K. 0.155* ± *0.01* *L. 0.065* ± *0.003 (vs. K p* < *0.001)* *M. 0.062* ± *0.001 (vs. K p* < *0.001)*	–
Liver					
Tamura et al. [27]	A. Control (n = 15) B. Free CDDP 8 mg/kg (n = 15) C. Microsphere-CDDP 30 mg/kg (n = 15) D. Microsphere-CDDP 35 mg/kg (n = 15)	A. 30.9 ± 0.5 B. 31.7 ± 1.4 C. 45.1 ± 2.2 (vs. A-B p < 0.001) D. 45.5 ± 2 .2 (vs. A-B p < 0.001)	–	A. 0.386 ± 0.036 B. 0.325.3 ± 0.042 C. 0.113 ± 0.014 (vs. A p < 0.001) D. 0.114.8 ± 0.014 (vs. A p < 0.001)	–
Wang et al. [78]	A. Control (n = 10) B. Free 5-FU 20 mg/kg (n = 10) C. Carrier erythrocyte-FU 20 mg/kg (n = 10)	A. 13 B. 20 C. 28 (NS)	n.a	n.a	n.a

All values in italics are values derived from figures and not exact numbers

5-FU = 5-fluorouracil; CDDP = cisplatin; DOX = doxorubicin; IRI = irinothecan; MIT = mitoxantrone; NS = not significant; PTX = paclitaxel; SD = standard deviation

^#^ Median survival times as reported by the studies' authors

**Table 8:** Study outcomes of studies using a hydrogel or a microsphere as a DDS

First author (ref)	Disease entity (Type of tumor cell line)	Number of cells administered to induce PM Time between tumor inoculation and start therapy (days)	Type and dosage of cytostatic agent administered	Experimental groups compared (n)	Results – Survival Median survival time (days) ^#^	Results – tumor load Mean total intraperitoneal tumor weight (gram ± SD), mean tumor volume (cm^3^ ± SD), mean number of tumor nodules ± SD, or signal intensity (photon counts ± SD)
**Hydrogels**						
Bae et al. [46]	Colon (CT-26luc)	1 × 10^5^ Inoculation period not stated	5-FU 100 mg/kg	A. Control (n = ?) B. 5-FU 100 mg/kg i.v. (n = ?) C. Free 5-FU 100 mg/kg i.p. (n = ?) D. 5-FU 100 mg/kg i.p. + Plu-CLA 20.8 mM (n = ?)	n.a	Significant inhibition of tumor growth (p < 0.05) (compared groups not stated)
Bae et al. [40]	Gastric (TMK1)	1 × 10^7^ 7 days	Docetaxel 10 mg/kg	A. Control (n = 5) B. Free DOC 10 mg/kg (n = 5) C. Hydrogel-DOC 10 mg/kg (n = 5)	A. 28 B. 31 C. 44 (vs. A-B p < 0.05)	A. 41.8 ± 6.47 cm^3^ B. 26.8 ± 5.99 cm^3^ C. 18.6 ± 4.67 cm^3^ (vs. A-B p < 0.05)
Chen et al. [47]	Colon (CT-26)	2 × 10^5^ 7 days	Doxorubicin 1 mg/kg	A. Control (n = 8) B. Blanc hydrogel (n = 8) C. Free DOX 1 mg/kg (n = 8) D. Hydrogel-DOX 1 mg/kg (n = 8)	A. 18 B. 19 C. 21 D. 29 (NS)	A. 2.50 ± 0.12 g B. 2.60 ± 0.08 g C. 1.13 ± 0.09 g D. 0.30 ± 0.03 g (vs. A-C p < 0.05) A. 2.16 ± 0.16 cm^3^ B. 2.70 ± 0.10 cm^3^ C. 1.46 ± 0.12 cm^3^ D. 0.46 ± 0.08 cm^3^ (vs. A-C p < 0.05)
Emoto et al. [68]	Gastric (MKN45P)	1 × 10^6^ 7 days	Cisplatin 1 mg/kg	A. Control (n = 6) B. Blanc hydrogel (n = 6) C. Free CDDP 1 mg/kg (n = 6) D. Hydrogel-CDDP 1 mg/kg (n = 6)	n.a	*A. 0.44* ± *0.42 g* *B. 0.26* ± *0.11 g* *C. 0.30* ± *0.13 g* *D. 0.13* ± *0.09 g (vs. A p* < *0.05)*
Fan et al. [37]	Colon (HCT)	5 × 10^6^ 10 days	Docetaxel + LL37 peptide 8–16 mg/kg	A. Control (n = 12) B. Blanc nanoparticle-hydrogel (n = 12) C. Free DOC 8 mg/kg (n = 12) D. Nanoparticle-hydrogel-DOC 16 mg/kg (n = 12) E. Free DOC + LL37 8 mg/kg (n = 12) F. Nanoparticle-hydrogel DOC + LL37 16 mg/kg (n = 12)	A. 29 B. 35 C. 45 D. 48 E. 49 F. 60 (vs. A-E p < 0.01)	A. 3.07 ± 0.39 g B. 3.13 ± 0.3 g C. 1.88 ± 0.16 g D. 1.66 ± 0.16 g E. 1.11 ± 0.10 g F. 0.61 ± 0.19 g (vs. A-E p < 0.01) A. 73.62 ± 8.68 B. 75.13 ± 4.64 C. 48.04 ± 7.18 D. 42.03 ± 6.36 E. 26.62 ± 4.72 F. 18.21 ± 1.92 (vs A-E p < 0.01)
Gong et al. [31]	Colon (CT-26)	IP 2 × 10^5^ 5 days	Paclitaxel 2–4 mg/kg FU 2–4 mg/kg	A. Control (n = 12) B. Blanc micelle-hydrogel (n = 12) C. Free FU 4 mg/kg (n = 12) D. Free PTX 4 mg/kg (n = 12) E. Free PTX 2 mg/kg + FU 2 mg/kg (n = 12) F. Micelle-PTX-hydrogel-FU (n = 12)	A. 23 B. 24 C. 30 D. 32 E. 35 F. 42 (NS)	*A. 3.2* ± *0.6 g* *B. 3.3* ± *0.5 g* *C. 1.2* ± *0.25 g* *D. 1.35* ± *0.2 g* *E. 0.95* ± *0.25 g* *F. 0.4* ± *0.35 g (vs. A-E p* < *0.001)* *A. 122* ± *52* *B. 126* ± *42* *C. 62* ± *17* *D. 64* ± *18* *E. 36* ± *16* *F. 16* ± *13* (vs. A-C p < 0.001)
Han et al. [41]	Gastric (44As3Luc)	IP 1 × 10^6^ 3 days	Docetaxel 2–8 mg/kg	A. Control (n = 10) B. Blanc hydrogel (n = 10) C. Free DOC i.v. 8 mg/kg (n = 10) D. Free DOC i.p. 8 mg/kg (n = 10) E. Hydrogel-DOC 2 mg/kg (n = 10) F. Hydrogel-DOC 8 mg/kg (n = 10)	A. 9.5 B. 17 C. 15 D. 42 E. 27.5 (vs. A-C p < 0.001) F. 102 (vs. A-C p < 0.001, vs. D p = 0.0068)	Day 8; day 14; day 28 A. 0.72; 0.96 cm^3^ B. 0.74; 1.71; 3.65 cm^3^ C. 0.81; 1.93 cm^3^ D. 0.10; 0.55; 2.94 cm^3^ E. 0.24; 0.47; 2.10 cm^3^ F. 0.0081; 0.022; 0.21 cm^3^ (NS)
Luo et al. [32]	Colon (CT-26)	IP 2 × 10^5^ 7 days	Paclitaxel 5 mg/kg Cisplatin 1 mg/kg 5-FU 20 mg/kg	A. Control (n = 10) B. Blanc hydrogel (n = 10) C. Free 5-FU 20 mg/kg, free PTX 5 mg/kg, free DDP 1 mg/kg (n = 10) D. Drug loaded hydrogel (n = 10)	A. 27 B. 26 C. 32 D. 36 (vs. A-C p < 0.05)	A. 88 ± 5.86 B.76 ± 5.86 C.29 ± 4.04 D. 14 ± 2.08 (vs. A-C p < 0.05)
Qian et al. [49]	Gastric (MKN45)	IP 5 × 10^6^ 14 days	Paclitaxel 8 mg/kg	A. Control (n = 5) B. Free PTX 8 mg/kg (n = 5) C. Nanoparticle-PTX 8 mg/kg (n = 5) D. Hydrogel-nanoparticle-PTX 8 mg/kg (n = 5)	n.a	*A. 0.77* ± *0.54 g* B. 0.63 ± 0.39 g C. 0.52 ± 0.25 g D. 0.33 ± 0.22 g (vs B and C p < 0.05) *A. 98* ± *19* B. 34.25 ± 11.67 C. 29.2 ± 4.87 D. 19.0 ± 8.0 (vs B and C p < 0.05)
Wang et al. [42]	Colon (CT-26)	IP 1 × 10^5^ 5 days	5-FU 25 mg/kg 2x (1 per week)	A. Control (n = 10) B. Blanc hydrogel (n = 10) C. Free 5-FU 25 mg/kg (n = 10) D. Hydrogel-5-FU 25 mg/kg (n = 10)	Survival rate (%) A. 62.5 B. 75 C. 62.5 D. 100 (vs. A-C p < 0.05)	A. 20.2 ± 10.08 B. 23.67 ± 6.98 C. 11.6 ± 3.8 D. 5.3 ± 4.04 (vs. A-C p < 0.05)
Xu et al. [48]	Colon (CT-26)	IP 1 × 10^5^ 5 days	Paclitaxel 30 mg/kg	A. Control (n = 8) B. Blanc hydrogel (n = 8) C. Free Taxol (n = 8) D. Hydrogel-PTX (n = 8)	n.a	A. 1.22 ± 0.83 g B. 1.24 g C. 0.76 ± 0.12 g D. 0.55 ± 0.14 g (vs. A p < 0.01, vs. C p < 0.05)
Yamashita et al. [69]	Gastric MKN45-Luc	IP 5 × 10^6^ 5 days	Cisplatin 5–10 mg/kg 2x	A. Control (n = 12) B. Blanc hydrogel granule (n = 12) C. Free CDDP 1 mg/kg (n = 12) D. Free CDDP 2 mg/kg (n = 12) E. Free CDDP 3 mg/kg (n = 12) F. Free CDDP 5 mg/kg (n = 12) G. Hydrogel granule-CDDP 5 mg/kg (n = 12) H. Hydrogel granule-CDDP 10 mg/kg (n = 12)	A.39 B. 34 C. 34 D. 41 E. 14 F. 10 G. 51 (vs. A p = 0.0012) H. 51 (vs. A p = 0.0012)	A. 61 ± 15 B. 48 ± 10 G. 21 ± 3 (vs A p < 0.05) H. 23 ± 2 (vs A p < 0.05)
Yu et al. [70]	Gastric (HSC44Luc)	IP 1 × 10^6^ 3 days	Paclitaxel 15–30 mg/kg	A. Control (n = 5) B. Blanc hydrogel (n = 5) C. Free PTX 30 mg/kg (n = 3) D. Hydrogel-PTX 15 mg/kg (n = 5)	n.a	A. 7.5 E + 06 photons/sec B. 7.0 E + 06 photons/sec C. 0.5 E + 0 photons/sec (vs A p < 0.05) D. 0.5 E + 0 photons/sec(vs A p < 0.05)
Yun et al. [33]	Colorectal (CT-26)	IP 2 × 10^5^ 7 days	5-FU 20 mg/kg Cisplatin 1 mg/kg	A. Control (n = 12) B. Blanc micelle-hydrogel (n = 12) C. Micelle-5-FU 20 mg/kg (n = 12) D. Hydrogel-CDDP 1 mg/kg (n = 12) E. Free 5-FU 20 mg/kg and free CDDP 1 mg/kg (n = 12) F. Hydrogel-CDDP + micelle-5-FU (n = 12)	A. 25 B. 26 C. 31 D. 33 E. 35 F. 43 (NS)	A. 53.83 ± 9.99 ( B. 52.67 ± 6.12 C. 22.5 ± 4.23 D. 23.22 ± 3.56 E. 18.16 ± 3.06 F. 10.33 ± 2.66 (vs. A-D p < 0.001, vs. E p < 0.05)
**Microspheres**						
Fan et al. [44]	Colorectal (CT-26)	IP 2 × 10^5^ 7 days	Docetaxel 4–8 mg/kg	A. Control (n = 8) B. Blanc microsphere (n = 8) C. Free DOC 4 mg/kg (n = 8) D. Microsphere-DOC 8 mg/kg (n = 8)	A. 23 B. 25 C. 29 D. 33 (vs. A-C p < 0.05)	*A. 160* ± *30* *B. 148* ± *23* *C. 80* ± *25* *D. 45* ± *5 (vs. A- C p* < *0.05)*
Fan et al. [43]	Colorectal (CT-26)	2 × 10^5^ 7 days	Docetaxel + Curcumin 8 mg/kg	A. Control (n = 12) B. Blanc microsphere (n = 12) C. Free DOC 8 mg/kg (n = 12) D. Microsphere-DOC (n = 12) E. Free DOC: curcumin (1:1) 8 mg/kg (n = 12) F. Microsphere-DOC + curcumin 8 mg/kg (n = 12)	A. 18 B. 20 C. 29 D. 39 E. 42 F. 48 (vs. A-E p < 0.05)	*A. 158* ± *30* *B. 143* ± *15* *C. 83* ± *9* *D. 66* ± *20* *E. 58* ± *10* *F. 32* ± *6 (vs. A-E p* < *0.05)*
Gunji et al. [45]	Colorectal (CT-26)	1 × 10^6^ 7 days	Cisplatin 10–20 mg/kg	Outcome tumor load: A. Control (n = 5) B. Blanc microsphere (n = 5) C. Free CDDP 10 mg/kg (n = 5) D. Microsphere-CDDP 10 mg/kg (n = 5) Outcome survival: A. Control (n = 6) B. Blanc microsphere (n = 6) C. Free CDDP 20 mg/kg (n = 6) D. Microsphere-CDDP 20 mg/kg (n = 6)	A. 18 B. 25 C. 40 ± 23 D. 74 ± 23 (vs. C p < 0.05)	A. 0.869 ± 0.452 g B. 1.070 ± 0.635 g C. 0.151 ± 0.066 g D. 0.108 ± 0.001 g (vs. A p < 0.001)
Inoue et al. [71]	Gastric (MKN45)	2 × 10^6^ 7 days	Floxuridine 1 mg/kg	A. Control (n = 6) B. Blanc microsphere (n = 6) C. Free FUDR bolus MTD (n = 6) D. Free FUDR bolus (n = 6) E. Microsphere-FUDR MTD (n = 6)	n.a	*Corrected for control group:* *B. 98* ± *23 g* *C. 45* ± *5 g (vs D p* < *0.05)* *D. 99* ± *38 g* *E. 10* ± *10 g*
Tamura et al. [27]	Gastric (H-145)	3 × 10^6^ 7 days	Cisplatin 20–40 mg/kg	A. Control (n = 9) B. Free CDDP 8 mg/kg (n = 9) C. Free CDDP 10 mg/kg (n = 9) D. Microsphere-CDDP 20 mg/kg (n = 9) E. Microsphere-CDDP 35 mg/kg (n = 9) F. Microsphere-CDDP 40 mg/kg (n = 9)	n.a	A. 0.75 ± 0.27 g B. 0.23 ± 0.10 g C. 0.16 ± 0.07 g D. 0.13 ± 0.04 g (vs. A p < 0.05) E. 0.13 ± 0.06 g F. 0.07 ± 0.02 g (vs. A p < 0.05)
Tamura et al. [27]	Liver (Li-7)	Number of cells not stated 8 days	Cisplatin 30–35 mg/kg	A. Control (n = 15) B. Free CDDP 8 mg/kg (n = 15) C. Microsphere-CDDP 30 mg/kg (n = 15) D. Microsphere-CDDP 35 mg/kg (n = 15)	A. 30.9 ± 0.5 B. 31.7 ± 1.4 C. 45.1 ± 2.2 (vs. A-B p < 0.001) D. 45.5 ± 2 .2 (vs. A-B p < 0.001)	A. 0.386 ± 0.036 cm^3^ B. 0.325.3 ± 0.042 cm^3^ C. 0.113 ± 0.014 cm^3^ (vs. A p < 0.001) D. 0.114.8 ± 0.014 cm^3^ (vs. A p < 0.001)

All values in italics are values derived from figures and not exact numbers

5-FU = 5-fluorouracil; CDDP = cisplatin; DDS = drug delivery systems; DOX = doxorubicin; IP = intraperitoneal; IV = intravenous; Luc = luciferase; NS = not significant; PTX = paclitaxel; SD = standard deviation, sec = second

^#^Median survival times as reported by the studies' authors

#### PM model of colorectal origin

Of the sixteen studies that used a PM model of colorectal origin, eleven studies had survival as an outcome parameter. These studies found that treatment with a cytostatic released from a DDS resulted in a higher MST, compared to treatment with a free cytostatic ("without DDS"), an empty DDS ("without cytostatic"), or no treatment. In five of the eleven studies, the difference was statistically significant, as displayed in Table 5. In the other studies, it was either not reported or the outcomes were not statistically different.

The longest absolute MST was found in the experimental study by Fan et al. [37]. In their study, animals inoculated with HCT-116 cells and treated with docetaxel co-encapsulated with LL37 peptide polymeric nanoparticles in a thermo-responsive hydrogel showed an MST of 60 days, whilst treatment without the addition of LL37 or with free docetaxel resulted in an MST of 48 and 45 days, respectively. The shortest absolute survival of animals receiving a cytostatic from a DDS was found by Cherukula et al., in which a lithocholic acid conjugated disulfide-linked polyethyleneimine micelle loaded with paclitaxel resulted in an MST of 27 days, compared to 21 days when treated with free paclitaxel [38].

#### PM model of gastric origin

Six out of the fourteen studies which had examined the PM models of gastric origin had survival as an outcome parameter. Similar results as in the PM colorectal models were found in the gastric models. A combination of a DDS loaded with a cytostatic always resulted in a longer MST; in five studies, this difference was statistically significant. The longest MST, of 126 days, was found by Kinoshita et al. Animals inoculated with OCUM-2MD3 cells and treated with nanoparticle albumin-bound loaded with paclitaxel had a longer survival compared to animals receiving a free drug (96 days) [39]. All results regarding PM models of gastric origin are shown in Table 6.

#### PM model of pancreatic- or liver origin

All seven studies using a pancreatic- or liver cancer cell line had survival as an outcome parameter. They all found that a cytostatic released from a DDS resulted in longer survival. In five studies, the difference was statistically significant compared to control and/or free drug. Table 7 displays all results regarding this PM model. The largest statistically significant difference regarding survival compared to the free drug group was found in the study of Tamura et al., in which microspheres loaded with 30 mg/kg and 35 mg/kg cisplatin were used to treat a PM model of liver cancer origin [27].

#### DDS: hydrogels

Most studies (8 out of 10) described an improved MST for the experimental groups with a cytostatic-loaded hydrogel system. When comparing survival of the DDS-cytostatic groups to the control groups without treatment, it was found that the difference in MST was statistically significant in six studies.

Five studies reported a statistically significant difference compared to the free drug group. Four of these used thermo-responsive hydrogels [37, 40–42], and one used a hyaluronic acid (HA) encapsulated PCEC microsphere [32].

In two studies (Han et al. & Bae et al.), a similar dose of docetaxel (8 mg/kg and 10 mg/kg respectively) was released from two types of thermo-responsive hydrogel systems, clearly demonstrating the potential for the hydrogel-based delivery of docetaxel to treat gastric PM [40, 41]. However, this model used two experimental parameters (cell line & inoculation time) which prevents a direct comparison between the two hydrogel systems. Fan et al. also chose docetaxel as their cytostatic agent, but the dose was higher and LL37 peptide was also added [37]. Wang et al. also reported the use of a thermo-responsive hydrogel, using a different type of cytostatic (5-FU) in a colorectal cancer model of PM [42]. In the fourth study, by Luo et al., a mixture was administrated of paclitaxel, cisplatin, and 5-FU, making it difficult to identify the most effective drug [32]. Table 8 displays all results regarding this DDS.

#### DDS: microspheres

All seven studies with survival as an outcome measure reported a statistically significant longer MST after treatment with cytostatic-loaded microspheres compared to the control group. In Table 8, the results of this DDS are shown. Three of these studies used a similar model for PM of colorectal origin based on the CT-26 cell line [43–45]. Despite the higher tumor cell number used to induce the PM, the microspheres used by Gunji et al. resulted in the best survival outcome (74 days vs. 18 and 40 for the control and free drug group respectively) [45]. The gelatin microspheres in their study used the carboxylic acid moiety to chelate cisplatin and prolong drug release. Both studies by Fan et al. used a lower cell number to induce the PM but the survival for the untreated control group appears similar to the control in the Gunji study. In the Fan et al. studies, the survival for the experimental group treated with the DDS appears lower [43, 44], indicating that the approach chosen by Gunji et al. might be better.

### Study outcomes: reduction of intraperitoneal tumor load

Another outcome parameter of interest here was the reduction in tumor load. This was determined as a reduction in tumor number, weight, or volume, or change in photon counts measured over time with an *in-vivo* imaging system. In most studies, the animals were sacrificed after a follow-up period and the intraperitoneal tumor load had been determined. The results regarding this outcome are described in Tables 5, 6, 7, and 8. Again, this outcome parameter is first explained per cell line and thereafter in light of the two most frequently used DDSs (hydrogel and microsphere).

#### PM model of colorectal origin

All sixteen studies with PM models of colorectal origin had tumor load as an outcome parameter and all demonstrated a reduction in tumor load in the experimental group treated with a DDS containing a cytostatic, compared to treatment with a free cytostatic, an empty DDS, or no treatment. In twelve studies, the tumor load was significantly lower in the group with cytostatic-loaded DDS compared to the group with the free drug. In one study, the difference was only statistically significant compared to the no treatment group, as displayed in Table 5.

#### PM model of gastric origin

In eleven out of the fourteen studies using a gastric cancer cell line, reduction of intraperitoneal tumor load was described as an outcome parameter and five studies described a significant difference between the group treated with cytostatic-loaded DDS compared and the group treated with free drug.

#### PM model of pancreatic- or liver origin

Only two of the seven studies using PM models of liver- or pancreas origin described tumor load as an outcome. None found a significant difference between animals treated with a cytostatic-loaded DDS compared to animals treated with a free drug.

#### DDSs: hydrogels

All DDS-hydrogel studies had the reduction of intraperitoneal tumor load as an outcome measure, as displayed in Table 8. Despite using different cytostatic agents and testing in various PM models, most (13 out of 14) studies using a hydrogel described a significant reduction in tumor load compared to the untreated group, whereas the majority (9 out of 14) described a significant reduction compared to IP administration of the free drug.

For instance, Bae et al. used the same thermo-responsive conjugated linoleic acid-coupled Pluronic F-127 hydrogel as a controlled release intraperitoneal delivery system in two studies. In the first [46], 5-FU was loaded in the hydrogel system and showed a significant inhibition of tumor growth at day 8 in a model for colorectal PM. In the second [40], the delivery of docetaxel was tested in a gastric PM model resulting in a significant inhibition of tumor growth. Wang et al. also tested the release of 5-FU from a thermo-responsive hydrogel (PECE) in the same model system for colorectal PM (CT-26 cell-line) [42]. However, due to the differences in dose, frequency of administration, and inoculation period, it is difficult to compare the suitability of the two respective hydrogel systems. Three studies investigated a hydrogel system on the same colorectal PM model with a single drug release: Chen et al. used doxorubicin [47], Fan et al. used docetaxel + LL37 [37], and Xu et al. used paclitaxel [48]. Since the outcome parameters for tumor load (tumor volume, number, and weight) were different in all three studies, it is difficult to choose the best type of cytostatic for this model. Another hydrogel system was investigated by Qian et al. [49]. They investigated a hydrogel-encapsulating red blood cell membrane nanoparticle using paclitaxel in a PM model of gastric cancer, yet no comparison can be made with other studies.

Three studies investigated the effect of different drugs simultaneously delivered from one hydrogel system, all on a colorectal PM model. Yun et al. investigated 5-FU & cisplatin [33], Gong et al. paclitaxel & 5-FU [31], and Luo et al. paclitaxel & cisplatin & 5-FU [32]. Tumor cell number, inoculation period, and total follow-up period were comparable between studies. Luo and Yun administered equivalent dosages of 5-FU and cisplatin, but Gong et al. chose a lower dosage, especially for 5-FU, which achieved a highly significant effect.

#### DDSs: microspheres

All studies using microspheres had the reduction in intraperitoneal tumor load as an outcome measure. However, only the two studies by Fan et al. reported a significant reduction in tumor load [43, 44]. In these studies, colorectal PM with identical cell number and inoculation period were treated with a cytostatic-loaded microsphere and compared to free drug. However, in the 2014 study, the docetaxel dose used in the free drug group (4 mg/kg) was only half that of the experimental group (8 mg/kg), making any comparison between these groups difficult. In the 2016 study, docetaxel was combined with curcumin to enhance the anti-tumor effect. Both studies resulted in comparable effects demonstrating the potential effects of docetaxel released from a microsphere-DDS in this PM model. These results are shown in Table 8.

## Discussion

This systematic review has provided an overview of laboratory animal studies in which a DDS containing a cytostatic agent was used to treat PM of colorectal-, gastric-, liver- and pancreatic origin. Two outcome parameters have been studied here: survival and reduction in intraperitoneal tumor load. Of the 35 studies, 23 had survival as an outcome parameter. In 15 (65%) of these studies, a statistically significant longer median survival time was described in animals treated with a DDS releasing a cytostatic, compared to treatment with a free cytostatic, an empty DDS, or no treatment. Furthermore, 25 studies investigated the effect on intraperitoneal tumor load; all studies reported the lowest tumor load in animals treated with a DDS containing a cytostatic agent. However, only in 6 (24%) of the studies this difference was statistically significant.

The results found here do indicate that delivering a DDS containing a cytostatic drug improves important clinical outcomes in an experimental setting, despite the fact that a large variety of DDSs, types of cytostatic agents, and types of cell lines used to induce PM in the laboratory animals were identified. The rationale behind treating PM using a DDS is that a higher intraperitoneal chemotherapy concentration can be administered for a prolonged period and with fewer systemic side effects. In clinic, the commonly used types of cytostatic agents to treat PM include paclitaxel, oxaliplatin, cisplatin, and mitomycin C. Because of the relatively small molecular weight (< 20 kDa) of these drugs, the systemic uptake and clearance are fast, so the presence of the drugs without a DDS in the peritoneal cavity is, therefore, too short for therapeutic purposes [50]. Pharmacokinetic animal studies have revealed that docetaxel and paclitaxel cleared within less than 24 h after intraperitoneal administration [51, 52]. Due to the controlled, regional releasing properties of e.g. a hydrogel, it is possible to prolong exposure of tumor nodules to cytostatics [50], which is expected to result in a higher decrease in tumor load. Other types of DDSs have different properties but with the same result, such as nano- or microparticles, liposomes, or microspheres; these can accumulate within tumor nodules through both enhanced permeability and the retention effect and, therefore, provide deeper penetration and prolonged exposure to cytostatics [53]. To improve tumor selectivity and therapeutic efficiency, tumor homing peptides such as iRGD or LL37 were linked to the nanoparticles [28, 37].

An important point to discuss is the low methodological quality of the included studies, something which seems to be inherent to animal research in general [54, 55]. As described in the results section, the reporting of methodology in the studies was poor, which resulted in an unclear risk of bias for the majority of the signaling items. In a similar vein, important methodological topics, such as randomization of animals over the treatment groups and information about the outcome assessor, tended not to be described according to the Animal Research: Reporting of In Vivo Experiments (ARRIVE) guidelines [56]. Therefore, the results need to be interpreted with caution. In addition, we must keep in mind that there is most likely an underreporting of studies in which the experimental DDSs had none or limited effect on outcomes of interest. Underreporting of negative results is inherent to research in general.

This systematic review has shown that administrating cytostatic drugs via a DDS might be useful in improving several important clinical outcomes such as survival or tumor load in an experimental animal setting. An important question is, however, how to translate these findings into clinical practice. First, it is important to select the patient group that will be best suited for treatment with a DDS containing a cytostatic agent. From clinical practice, in the current curative treatment option of PM, it is recognized that removing all macroscopic tumor nodules during cytoreductive surgery is the most important prognostic determinant. In none of the studies included here was cytoreductive surgery performed before the DDS was administered. As the inoculation period was relatively short for most studies, the resulting limited peritoneal tumor load mimics the situation directly after the curative-intent cytoreductive surgery procedure [57]. Replacing the HIPEC procedure by administrating the cytostatic-loaded DDS could be considered, as this has the advantage of prolonged intra-abdominal exposure time to the cytostatic. Treatment with a DDS containing a cytostatic agent might be effective in improving the MST in a patient group with limited PM after cytoreductive surgery.

For the patients with an advanced stage of PM who are not eligible for curative-intent CRS treatment, the DDS administration could be considered a palliative option to prolong life.

In this respect, Lu et al. investigated the effect of a cytostatic-loaded DDS on both early- and late-stage PM of pancreatic origin. The MST in the early-stage group of animals treated with a DDS containing a cytostatic was much longer than in the late-stage group. However, a statistically significant longer MST was also found in the late-stage animals treated with a paclitaxel-loaded DDS compared to the free paclitaxel suggesting that DDS loaded with cytostatic might indeed have an additional value also in the palliative setting [58]. The second point is how clinically relevant the improved outcomes are for the patient. For example, does the improvement in survival translate into a prolonged life expectancy of days, weeks, or maybe even months?

It is notable that none of these studies described complications or side effects observed in the animals after administration, apart from changes in body weight in the first days after administrating the DDS in some of the studies. It seems, however, highly unlikely that there were no such effects at all. To outweigh the benefit of the improved clinical outcomes against the possible harm caused by the treatment with a cytostatic-loaded DDS, it is important to gain greater insight into potential complications before doing research with larger laboratory animals and clinical trials. To the best of our knowledge, no clinical trials have been published yet in which a certain type of DDS was used to treat PM originating from colorectal-, gastric-, pancreatic- or liver cancer. However, several phase I studies primarily investigating the safety, tolerability, and pharmacokinetics of a nanoparticle albumin-bound paclitaxel have been published, and those have revealed the safety of this cytostatic-loaded DDS [59, 60].

An important finding of this systematic review is that quite a few experimental studies have already been conducted into this topic, yet none have used the same experimental setting. Indeed, the large variety in choice of type of DDS, type, and dose of cytostatic, or cell line used to induce PM makes it difficult to determine the optimal treatment. Therefore, we encourage collaboration between researchers and clinical physicians treating patients with PM when designing new studies so as to ensure that clinical relevance is taken into account.

The gap between clinical practice and preclinical experiments might be shortened by using organoids [61]. Organoids, which are derived from tissue and ascites samples taken from patients with PM, capture the functional heterogeneity and genetic phenotypic characteristics of PM. Organoid technology makes it possible to create more realistic PM models to test DDSs, as the therapeutic response to these models would be more similar to the response observed in the clinic.

From a more technical point of view, a more rational approach is needed to the design of the DDS and the type of drug than the current ad hoc approach in the preclinical experimental literature. In clinical i.p. chemotherapy, a handful of chemotherapeutics are used to treat PM from the different gastro-intestinal origins [24, 62–64]. However, that does not mean that one drug alone is effective in treating every type of PM. In clinical practice, specific drugs are preferred for the treatment of each type of PM: for instance, mitomycin C for colorectal and appendicular PM, taxanes (paclitaxel and docetaxel) for gastric and ovarian PM, irinotecan and 5-FU for colorectal PM, and platinum-based agents for colorectal, gastric, and ovarian PM. In this context, for some of the studies included here, there appears to be a mismatch in the selected drug and model system.

A rational design should also be applied to the combination of DDSs and cytostatic drugs. For example, hydrophobic drugs such as taxanes and irinotecan are incorporated in the hydrophobic polymer domains of thermo-reversible hydrogels or micro- and nanoparticles, resulting in increased retention. More hydrophilic compounds such as mitomycin C, or 5-FU are retained less efficiently in similar systems. For platinum-based compounds, metal-complexation strategies could be used to link the drug to the carboxylic groups of the DDS.

To realize drug release rates and concentrations that are expected to be safe and effective in the clinical practice research should be based on pharmacokinetic data from previous clinical studies. A thorough understanding of the interactions between the drug and DDS, and resulting drug retention, should be utilized.

This study is the first systematic review to comprehensively describe the effectiveness of DDSs for the treatment of PM of gastro-intestinal origin in experimental studies. Previously, van Oudheusden et al. had summarized the available from studies up to 2015 but did not systematically describe the results on survival and tumor-load [65]. The most important limitation of our review is that it was impossible to conduct a meta-analysis because of the large heterogeneity between the studies and their rather poor methodological quality and reporting. All of the studies found the longest survival time for animals treated with a cytostatic-loaded DDS. Also, none described a higher tumor load in the group treated with a cytostatic-loaded DDS compared to a free cytostatic. These findings perhaps indicate publication bias. It is estimated that only half of all laboratory animal research is published, with lack of statistical significance often the most important reasons for non-publication [66]. Thus, negative results in animal experimental studies have less chance of being published. Possible solutions for this problem have already been suggested, for example special journals for ‘negative’ results, or initially submitting a manuscript but without any results [67]. Adopting such measures might give a more realistic idea of how effective certain novel treatment modalities are before they are considered for implementation in the clinic. Another limitation of this review is that studies published before the year 2000 were excluded. The rationales behind this are that we think that the most interesting DDS developments took place within the last two decades. Also, promising DDS developed before this period would most probably have already resulted in clinical implementation. Therefore, we aimed on focusing only on more recent studies. The final possible limitation of this review is that it has only included studies regarding PM from a gastro-intestinal origin and thus does not provide a complete overview of all available PM studies is given.

## Conclusion

Based on the results presented, the delivery of a cytostatic with a DDS might lead to a higher median survival time and a lower intraperitoneal tumor-load compared to no treatment or treatment with free cytostatics or an empty DDS. Nevertheless, due to the poor methodological quality and reporting, any interpretation of results needs to be done with caution. The large variety in experimental setup makes it impossible to identify the optimal combination of DDSs and type of cytostatic for a specific tumor origin. Future studies should thus focus more on collaborating with clinical experts to design the studies in such way that their results would be clinically relevant. Greater attention should also be paid to the methodological quality and reporting of the experiments. Similarly, any complications and side effects of the administered novel therapy should be reported as an outcome. Additionally, more effort should be put into having animal studies with negative results published as well, so as to avoid treatment effects being overestimated. Finally, standardization of the experimental designs should be taken into account. When designing a new study in which a novel DDS is investigated, several items have to be considered for design in a standardized manner, such as the choice of animal species, cell line, and number of cells used to induce PM, inoculation period, and therapeutic dose of the cytostatic agent.

## Data Availability

The datasets generated during and/or analyzed during the current study are available from the corresponding author on reasonable request.

## PubMed search

((((((((((((((Peritoneal cancer*[tiab]) OR Peritoneal carcinomatos*[tiab]) OR Peritoneal metastas*[tiab]) OR Intraperitoneal tumo*[tiab]) OR Intra-peritoneal tumo*[tiab]) OR Peritoneal tumo*[tiab]) OR Peritoneal malignanc*[tiab]) OR Peritoneal surface malignanc*[tiab]) OR Peritoneal neoplasm*[tiab])) OR "Peritoneal Neoplasms"[Mesh]))) AND

((((((((((((((((((((Hydrogel*[tiab]) OR Nanoparticle*[tiab]) OR Nano-particle*[tiab]) OR Micelle*[tiab]) OR Microsphere*[tiab]) OR Micro-sphere*[tiab]) OR Microparticle*[tiab]) OR Micro-particle*[tiab]) OR Liposome*[tiab]) OR Nanocarrier*[tiab]) OR Dendrimer*[tiab]) OR Polymer*[tiab]) OR Carrier*[tiab]) OR Microcapsule*[tiab]) OR Micro-capsule*[tiab]) OR Drug delivery system*[tiab]) OR Drug targeting*[tiab])) OR "Drug Delivery Systems"[Mesh]))) AND

(((((("animal experimentation"[MeSH] OR "models, animal"[MeSH] OR "invertebrates"[MeSH] OR "Animals"[Mesh:noexp] OR "animal population groups"[MeSH] OR "chordata"[MeSH Terms:noexp] OR "chordata, nonvertebrate"[MeSH] OR "vertebrates"[MeSH] OR "amphibians"[MeSH] OR "birds"[MeSH] OR "fishes"[MeSH] OR "reptiles"[MeSH] OR "mammals"[MeSH Terms:noexp] OR "primates"[MeSH Terms:noexp] OR "artiodactyla"[MeSH] OR "carnivora"[MeSH] OR "cetacea"[MeSH] OR "chiroptera"[MeSH] OR "elephants"[MeSH] OR "hyraxes"[MeSH] OR "insectivora"[MeSH] OR "lagomorpha"[MeSH] OR "marsupialia"[MeSH] OR "monotremata"[MeSH] OR "perissodactyla"[MeSH] OR "rodentia"[MeSH] OR "scandentia"[MeSH] OR "sirenia"[MeSH] OR "xenarthra"[MeSH] OR "haplorhini"[MeSH Terms:noexp] OR "strepsirhini"[MeSH] OR "platyrrhini"[MeSH] OR "tarsii"[MeSH] OR "catarrhini"[MeSH] OR "cercopithecidae"[MeSH] OR "hylobatidae"[MeSH] OR "hominidae"[MeSH Terms] OR "gorilla gorilla"[MeSH] OR "pan paniscus"[MeSH] OR "pan troglodytes"[MeSH] OR "pongo pygmaeus"[MeSH]) OR ((animals[tiab] OR animal[tiab] OR mice[tiab] OR mus[tiab] OR mouse[tiab] OR murine[tiab] OR woodmouse[tiab] OR rats[tiab] OR rat[tiab] OR murinae[tiab] OR muridae[tiab] OR cottonrat[tiab] OR cottonrats[tiab] OR hamster[tiab] OR hamsters[tiab] OR cricetinae[tiab] OR rodentia[tiab] OR rodent[tiab] OR rodents[tiab] OR pigs[tiab] OR pig[tiab] OR swine[tiab] OR swines[tiab] OR piglets[tiab] OR piglet[tiab] OR boar[tiab] OR boars[tiab] OR "sus scrofa"[tiab] OR ferrets[tiab] OR ferret[tiab] OR polecat[tiab] OR polecats[tiab] OR "mustela putorius"[tiab] OR "guinea pigs"[tiab] OR "guinea pig"[tiab] OR cavia[tiab] OR callithrix[tiab] OR marmoset[tiab] OR marmosets[tiab] OR cebuella[tiab] OR hapale[tiab] OR octodon[tiab] OR chinchilla[tiab] OR chinchillas[tiab] OR gerbillinae[tiab] OR gerbil[tiab] OR gerbils[tiab] OR jird[tiab] OR jirds[tiab] OR merione[tiab] OR meriones[tiab] OR rabbits[tiab] OR rabbit[tiab] OR hares[tiab] OR hare[tiab] OR diptera[tiab] OR flies[tiab] OR fly[tiab] OR dipteral[tiab] OR drosophila[tiab] OR drosophilidae[tiab] OR cats[tiab] OR cat[tiab] OR carus[tiab] OR felis[tiab] OR nematoda[tiab] OR nematode[tiab] OR nematodes[tiab] OR sipunculida[tiab] OR dogs[tiab] OR dog[tiab] OR canine[tiab] OR canines[tiab] OR canis[tiab] OR sheep[tiab] OR sheeps[tiab] OR mouflon[tiab] OR mouflons[tiab] OR ovis[tiab] OR goats[tiab] OR goat[tiab] OR capra[tiab] OR capras[tiab] OR rupicapra[tiab] OR rupicapras[tiab] OR chamois[tiab] OR haplorhini[tiab] OR monkey[tiab] OR monkeys[tiab] OR anthropoidea[tiab] OR anthropoids[tiab] OR saguinus[tiab] OR tamarin[tiab] OR tamarins[tiab] OR leontopithecus[tiab] OR hominidae[tiab] OR ape[tiab] OR apes[tiab] OR "pan paniscus"[tiab] OR bonobo[tiab] OR bonobos[tiab] OR "pan troglodytes"[tiab] OR gibbon[tiab] OR gibbons[tiab] OR siamang[tiab] OR siamangs[tiab] OR nomascus[tiab] OR symphalangus[tiab] OR chimpanzee[tiab] OR chimpanzees[tiab] OR prosimian[tiab] OR prosimians[tiab] OR "bush baby"[tiab] OR bush babies[tiab] OR galagos[tiab] OR galago[tiab] OR pongidae[tiab] OR gorilla[tiab] OR gorillas[tiab] OR "pongo pygmaeus"[tiab] OR orangutan[tiab] OR orangutans[tiab] OR lemur[tiab] OR lemurs[tiab] OR lemuridae[tiab] OR horse[tiab] OR horses[tiab] OR equus[tiab] OR cow[tiab] OR calf[tiab] OR bull[tiab] OR chicken[tiab] OR chickens[tiab] OR gallus[tiab] OR quail[tiab] OR bird[tiab] OR birds[tiab] OR quails[tiab] OR poultry[tiab] OR poultries[tiab] OR fowl[tiab] OR fowls[tiab] OR reptile[tiab] OR reptilia[tiab] OR reptiles[tiab] OR snakes[tiab] OR snake[tiab] OR lizard[tiab] OR lizards[tiab] OR alligator[tiab] OR alligators[tiab] OR crocodile[tiab] OR crocodiles[tiab] OR turtle[tiab] OR turtles[tiab] OR amphibian[tiab] OR amphibians[tiab] OR amphibia[tiab] OR frog[tiab] OR frogs[tiab] OR bombina[tiab] OR salientia[tiab] OR toad[tiab] OR toads[tiab] OR "epidalea calamita"[tiab] OR salamander[tiab] OR salamanders[tiab] OR eel[tiab] OR eels[tiab] OR fish[tiab] OR fishes[tiab] OR pisces[tiab] OR catfish[tiab] OR catfishes[tiab] OR siluriformes[tiab] OR arius[tiab] OR heteropneustes[tiab] OR sheatfish[tiab] OR perch[tiab] OR perches[tiab] OR percidae[tiab] OR perca[tiab] OR trout[tiab] OR trouts[tiab] OR char[tiab] OR chars[tiab] OR salvelinus[tiab] OR minnow[tiab] OR cyprinidae[tiab] OR carps[tiab] OR carp[tiab] OR zebrafish[tiab] OR zebrafishes[tiab] OR goldfish[tiab] OR goldfishes[tiab] OR guppy[tiab] OR guppies[tiab] OR chub[tiab] OR chubs[tiab] OR tinca[tiab] OR barbels[tiab] OR barbus[tiab] OR pimephales[tiab] OR promelas[tiab] OR "poecilia reticulata"[tiab] OR mullet[tiab] OR mullets[tiab] OR eel[tiab] OR eels[tiab] OR seahorse[tiab] OR seahorses[tiab] OR mugil curema[tiab] OR atlantic cod[tiab] OR shark[tiab] OR sharks[tiab] OR catshark[tiab] OR anguilla[tiab] OR salmonid[tiab] OR salmonids[tiab] OR whitefish[tiab] OR whitefishes[tiab] OR salmon[tiab] OR salmons[tiab] OR sole[tiab] OR solea[tiab] OR lamprey[tiab] OR lampreys[tiab] OR pumpkinseed[tiab] OR sunfish[tiab] OR sunfishes[tiab] OR tilapia[tiab] OR tilapias[tiab] OR turbot[tiab] OR turbots[tiab] OR flatfish[tiab] OR flatfishes[tiab] OR sciuridae[tiab] OR squirrel[tiab] OR squirrels[tiab] OR chipmunk[tiab] OR chipmunks[tiab] OR suslik[tiab] OR susliks[tiab] OR vole[tiab] OR voles[tiab] OR lemming[tiab] OR lemmings[tiab] OR muskrat[tiab] OR muskrats[tiab] OR lemmus[tiab] OR otter[tiab] OR otters[tiab] OR marten[tiab] OR martens[tiab] OR martes[tiab] OR weasel[tiab] OR badger[tiab] OR badgers[tiab] OR ermine[tiab] OR mink[tiab] OR minks[tiab] OR sable[tiab] OR sables[tiab] OR gulo[tiab] OR gulos[tiab] OR wolverine[tiab] OR wolverines[tiab] OR mustela[tiab] OR llama[tiab] OR llamas[tiab] OR alpaca[tiab] OR alpacas[tiab] OR camelid[tiab] OR camelids[tiab] OR guanaco[tiab] OR guanacos[tiab] OR chiroptera[tiab] OR chiropteras[tiab] OR bat[tiab] OR bats[tiab] OR fox[tiab] OR foxes[tiab] OR iguana[tiab] OR iguanas[tiab] OR xenopus laevis[tiab] OR parakeet[tiab] OR parakeets[tiab] OR parrot[tiab] OR parrots[tiab] OR donkey[tiab] OR donkeys[tiab] OR mule[tiab] OR mules[tiab] OR zebra[tiab] OR zebras[tiab] OR shrew[tiab] OR shrews[tiab] OR bison[tiab] OR bisons[tiab] OR buffalo[tiab] OR buffaloes[tiab] OR deer[tiab] OR deers[tiab] OR bear[tiab] OR bears[tiab] OR panda[tiab] OR pandas[tiab] OR "wild hog"[tiab] OR "wild boar"[tiab] OR fitchew[tiab] OR fitch[tiab] OR beaver[tiab] OR beavers[tiab] OR jerboa[tiab] OR jerboas[tiab] OR capybara[tiab] OR capybaras[tiab] OR canine [tiab] OR bovine [tiab] OR porcine [tiab] OR hog [tiab] OR hogs [tiab]) NOT medline[sb])))))).

## Embase search

1. exp peritoneum cancer/
2. (peritoneal cancer* or peritoneal carcinomatos* or peritoneal metastas* or intraperitoneal tumo* or peritoneal tumo* or peritoneal malignanc* or peritoneal surface malignanc* or peritoneal neoplasm*).ab,kw,ti.
3. 1 or 2
4. exp drug delivery system/
5. (hydrogel* or nanoparticle* or micelle* or microsphere* or microparticle* or liposome* or nanocarrier* or dendrimer* or polymer* or carrier* or microcapsule* or drug delivery system* or drug targeting*).ab,kw,ti.
6. 4 or 5
7. exp animal experiment/ or exp animal model/ or exp experimental animal/ or exp transgenic animal/ or exp male animal/ or exp female animal/ or exp juvenile animal/ or animal/ or chordata/ or vertebrate/ or tetrapod/ or exp fish/ or amniote/ or exp amphibia/ or mammal/ or exp reptile/ or exp sauropsid/ or therian/ or exp monotremate/ or placental mammals/ or exp marsupial/ or Euarchontoglires/ or exp Afrotheria/ or exp Boreoeutheria/ or exp Laurasiatheria/ or exp Xenarthra/ or primate/ or exp Dermoptera/ or exp Glires/ or exp Scandentia/ or Haplorhini/ or exp prosimian/ or simian/ or exp tarsiiform/ or Catarrhini/ or exp Platyrrhini/ or ape/ or exp Cercopithecidae/ or hominid/ or exp hylobatidae/ or exp chimpanzee/ or exp gorilla/ or exp orang utan/ or (animal or animals or pisces or fish or fishes or catfish or catfishes or sheatfish or silurus or arius or heteropneustes or clarias or gariepinus or fathead minnow or fathead minnows or pimephales or promelas or cichlidae or trout or trouts or char or chars or salvelinus or salmo or oncorhynchus or guppy or guppies or millionfish or poecilia or goldfish or goldfishes or carassius or auratus or mullet or mullets or mugil or curema or shark or sharks or cod or cods or gadus or morhua or carp or carps or cyprinus or carpio or killifish or eel or eels or anguilla or zander or sander or lucioperca or stizostedion or turbot or turbots or psetta or flatfish or flatfishes or plaice or pleuronectes or platessa or tilapia or tilapias or oreochromis or sarotherodon or common sole or dover sole or solea or zebrafish or zebrafishes or danio or rerio or seabass or dicentrarchus or labrax or morone or lamprey or lampreys or petromyzon or pumpkinseed or pumpkinseeds or lepomis or gibbosus or herring or clupea or harengus or amphibia or amphibian or amphibians or anura or salientia or frog or frogs or rana or toad or toads or bufo or xenopus or laevis or bombina or epidalea or calamita or salamander or salamanders or newt or newts or triturus or reptilia or reptile or reptiles or bearded dragon or pogona or vitticeps or iguana or iguanas or lizard or lizards or anguis fragilis or turtle or turtles or snakes or snake or aves or bird or birds or quail or quails or coturnix or bobwhite or colinus or virginianus or poultry or poultries or fowl or fowls or chicken or chickens or gallus or zebra finch or taeniopygia or guttata or canary or canaries or serinus or canaria or parakeet or parakeets or grasskeet or parrot or parrots or psittacine or psittacines or shelduck or tadorna or goose or geese or branta or leucopsis or woodlark or lullula or flycatcher or ficedula or hypoleuca or dove or doves or geopelia or cuneata or duck or ducks or greylag or graylag or anser or harrier or circus pygargus or red knot or great knot or calidris or canutus or godwit or limosa or lapponica or meleagris or gallopavo or jackdaw or corvus or monedula or ruff or philomachus or pugnax or lapwing or peewit or plover or vanellus or swan or cygnus or columbianus or bewickii or gull or chroicocephalus or ridibundus or albifrons or great tit or parus or aythya or fuligula or streptopelia or risoria or spoonbill or platalea or leucorodia or blackbird or turdus or merula or blue tit or cyanistes or pigeon or pigeons or columba or pintail or anas or starling or sturnus or owl or athene noctua or pochard or ferina or cockatiel or nymphicus or hollandicus or skylark or alauda or tern or sterna or teal or crecca or oystercatcher or haematopus or ostralegus or shrew or shrews or sorex or araneus or crocidura or russula or european mole or talpa or chiroptera or bat or bats or eptesicus or serotinus or myotis or dasycneme or daubentonii or pipistrelle or pipistrellus or cat or cats or felis or catus or feline or dog or dogs or canis or canine or canines or otter or otters or lutra or badger or badgers or meles or fitchew or fitch or foumart or foulmart or ferrets or ferret or polecat or polecats or mustela or putorius or weasel or weasels or fox or foxes or vulpes or common seal or phoca or vitulina or grey seal or halichoerus or horse or horses or equus or equine or equidae or donkey or donkeys or mule or mules or pig or pigs or swine or swines or hog or hogs or boar or boars or porcine or piglet or piglets or sus or scrofa or llama or llamas or lama or glama or deer or deers or cervus or elaphus or cow or cows or bos taurus or bos indicus or bovine or bull or bulls or cattle or bison or bisons or sheep or sheeps or ovis aries or ovine or lamb or lambs or mouflon or mouflons or goat or goats or capra or caprine or chamois or rupicapra or leporidae or lagomorpha or lagomorph or rabbit or rabbits or oryctolagus or cuniculus or laprine or hares or lepus or rodentia or rodent or rodents or murinae or mouse or mice or mus or musculus or murine or woodmouse or apodemus or rat or rats or rattus or norvegicus or guinea pig or guinea pigs or cavia or porcellus or hamster or hamsters or mesocricetus or cricetulus or cricetus or gerbil or gerbils or jird or jirds or meriones or unguiculatus or jerboa or jerboas or jaculus or chinchilla or chinchillas or beaver or beavers or castor fiber or castor canadensis or sciuridae or squirrel or squirrels or sciurus or chipmunk or chipmunks or marmot or marmots or marmota or suslik or susliks or spermophilus or cynomys or cottonrat or cottonrats or sigmodon or vole or voles or microtus or myodes or glareolus or primate or primates or prosimian or prosimians or lemur or lemurs or lemuridae or loris or bush baby or bush babies or bushbaby or bushbabies or galago or galagos or anthropoidea or anthropoids or simian or simians or monkey or monkeys or marmoset or marmosets or callithrix or cebuella or tamarin or tamarins or saguinus or leontopithecus or squirrel monkey or squirrel monkeys or saimiri or night monkey or night monkeys or owl monkey or owl monkeys or douroucoulis or aotus or spider monkey or spider monkeys or ateles or baboon or baboons or papio or rhesus monkey or macaque or macaca or mulatta or cynomolgus or fascicularis or green monkey or green monkeys or chlorocebus or vervet or vervets or pygerythrus or hominoidea or ape or apes or hylobatidae or gibbon or gibbons or siamang or siamangs or nomascus or symphalangus or hominidae or orangutan or orangutans or pongo or chimpanzee or chimpanzees or pan troglodytes or bonobo or bonobos or pan paniscus or gorilla or gorillas or troglodytes).ti,ab.
8. 3 and 6 and 7.

## References

[1] Desai JP, Moustarah F (2019) Cancer, Peritoneal Metastasis. StatPearls [Internet] 31082158

[2] Lurvink RJ, Bakkers C, Rijken A, van Erning FN, Nienhuijs SW, Burger JW (2021). Increase in the incidence of synchronous and metachronous peritoneal metastases in patients with colorectal cancer: a nationwide study. Eur J Surg Oncol.

[3] Jayne DG, Fook S, Loi C, Seow-Choen F (2002). Peritoneal carcinomatosis from colorectal cancer. Br J Surg.

[4] Thomassen I, van Gestel YR, van Ramshorst B, Luyer MD, Bosscha K, Nienhuijs SW (2014). Peritoneal carcinomatosis of gastric origin: a population-based study on incidence, survival and risk factors. Int J Cancer.

[5] Thomassen I, Lemmens VE, Nienhuijs SW, Luyer MD, Klaver YL, de Hingh IH (2013). Incidence, prognosis, and possible treatment strategies of peritoneal carcinomatosis of pancreatic origin: a population-based study. Pancreas.

[6] Rijken A, Bakkers C, van Erning FN, van der Geest LG, de Vos-Geelen J, Besselink MG (2021). Incidence, treatment, and survival of synchronous peritoneal metastases in pancreatic cancer: update of a nationwide cohort. Pancreas.

[7] Rijken A, Lurvink RJ, Luyer MDP, Nieuwenhuijzen GAP, van Erning FN, van Sandick JW (2021). The burden of peritoneal metastases from gastric cancer: a systematic review on the incidence, risk factors and survival. J Clin Med.

[8] Kranenburg O, van der Speeten K, de Hingh I (2021). Peritoneal metastases from colorectal cancer: defining and addressing the challenges. Front Oncol.

[9] Sadeghi B, Arvieux C, Glehen O, Beaujard AC, Rivoire M, Baulieux J (2000). Peritoneal carcinomatosis from non-gynecologic malignancies. Cancer.

[10] Toussaint L, Farinha HT, Barras J-L, Demartines N, Sempoux C, Hübner M (2021). Histological regression of gastrointestinal peritoneal metastases after systemic chemotherapy. Pleura Peritoneum..

[11] Franko J, Shi Q, Goldman CD, Pockaj BA, Nelson GD, Goldberg RM (2012). Treatment of colorectal peritoneal carcinomatosis with systemic chemotherapy: a pooled analysis of north central cancer treatment group phase III trials N9741 and N9841. J Clin Oncol.

[12] Verwaal VJ, Ruth SV, Bree ED, Slooten GW, Tinteren HV, Boot H (2003). Randomized trial of cytoreduction and hyperthermic intraperitoneal chemotherapy versus systemic chemotherapy and palliative surgery in patients with peritoneal carcinomatosis of colorectal cancer. J Clin Oncol.

[13] Glehen O, Gilly FN, Boutitie F, Bereder JM, Quenet F, Sideris L (2010). Toward curative treatment of peritoneal carcinomatosis from nonovarian origin by cytoreductive surgery combined with perioperative intraperitoneal chemotherapy. Cancer.

[14] Quénet F, Elias D, Roca L, Goéré D, Ghouti L, Pocard M (2021). Cytoreductive surgery plus hyperthermic intraperitoneal chemotherapy versus cytoreductive surgery alone for colorectal peritoneal metastases (PRODIGE 7): a multicentre, randomised, open-label, phase 3 trial. Lancet Oncol.

[15] Klaver YL, Lemmens VE, Creemers GJ, Rutten HJ, Nienhuijs SW, de Hingh IH (2011). Population-based survival of patients with peritoneal carcinomatosis from colorectal origin in the era of increasing use of palliative chemotherapy. Ann Oncol.

[16] Razenberg LG, van Gestel YR, Creemers GJ, Verwaal VJ, Lemmens VE, de Hingh IH (2015). Trends in cytoreductive surgery and hyperthermic intraperitoneal chemotherapy for the treatment of synchronous peritoneal carcinomatosis of colorectal origin in the Netherlands. Eur J Surg Oncol.

[17] Sun BJ, Lee B (2022). Review of regional therapies for gastric cancer with peritoneal metastases. Cancers.

[18] Brind’Amour A, Webb M, Parapini M, Sidéris L, Segedi M, Chung SW (2021). The role of intraperitoneal chemotherapy in the surgical management of pancreatic ductal adenocarcinoma: a systematic review. Clin Exp Metastasis.

[19] Grass F, Vuagniaux A, Teixeira-Farinha H, Lehmann K, Demartines N, Hübner M (2017). Systematic review of pressurized intraperitoneal aerosol chemotherapy for the treatment of advanced peritoneal carcinomatosis. Br J Surg.

[20] Willaert W, Sessink P, Ceelen W (2017). Occupational safety of pressurized intraperitoneal aerosol chemotherapy (PIPAC). Pleura Peritoneum.

[21] Sgarbura O, Hübner M, Alyami M, Eveno C, Gagnière J, Pache B (2019). Oxaliplatin use in pressurized intraperitoneal aerosol chemotherapy (PIPAC) is safe and effective: a multicenter study. Eur J Surg Oncol.

[22] Lurvink RJ, Van der Speeten K, Rovers KP, de Hingh I (2021). The emergence of pressurized intraperitoneal aerosol chemotherapy as a palliative treatment option for patients with diffuse peritoneal metastases: a narrative review. J Gastrointest Oncol.

[23] Rovers KP, Wassenaar ECE, Lurvink RJ, Creemers GM, Burger JWA, Los M (2021). Pressurized intraperitoneal aerosol chemotherapy (oxaliplatin) for unresectable colorectal peritoneal metastases: a multicenter, single-arm, phase II trial (CRC-PIPAC). Ann Surg Oncol.

[24] Valle SJ, Alzahrani NA, Liauw W, Sugarbaker PH, Bhatt A, Morris DL (2016). Hyperthermic intraperitoneal chemotherapy (HIPEC) methodology, drugs and bidirectional chemotherapy. Indian J Surg Oncol.

[25] Ouzzani M, Hammady H, Fedorowicz Z, Elmagarmid A (2016). Rayyan-a web and mobile app for systematic reviews. Syst Rev.

[26] Hooijmans CR, Rovers MM, de Vries RB, Leenaars M, Ritskes-Hoitinga M, Langendam MW (2014). SYRCLE's risk of bias tool for animal studies. BMC Med Res Methodol.

[27] Tamura T, Fujita F, Tanimoto M, Koike M, Suzuki A, Fujita M (2002). Anti-tumor effect of intraperitoneal administration of cisplatin-loaded microspheres to human tumor xenografted nude mice. J Control Release.

[28] Simón-Gracia L, Hunt H, Scodeller P, Gaitzsch J, Kotamraju VR, Sugahara KN (2016). iRGD peptide conjugation potentiates intraperitoneal tumor delivery of paclitaxel with polymersomes. Biomaterials.

[29] Tsai M, Lu Z, Wang J, Yeh TK, Wientjes MG, Au JL (2007). Effects of carrier on disposition and antitumor activity of intraperitoneal Paclitaxel. Pharm Res.

[30] Emoto S, Yamaguchi H, Kishikawa J, Yamashita H, Ishigami H, Kitayama J (2012). Antitumor effect and pharmacokinetics of intraperitoneal NK105, a nanomicellar paclitaxel formulation for peritoneal dissemination. Cancer Sci.

[31] Gong C, Wang C, Wang Y, Wu Q, Zhang D, Luo F (2012). Efficient inhibition of colorectal peritoneal carcinomatosis by drug loaded micelles in thermosensitive hydrogel composites. Nanoscale.

[32] Luo J, Wu Z, Lu Y, Xiong K, Wen Q, Zhao L (2020). Intraperitoneal administration of biocompatible hyaluronic acid hydrogel containing multi-chemotherapeutic agents for treatment of colorectal peritoneal carcinomatosis. Int J Biol Macromol.

[33] Yun Q, Wang SS, Xu S, Yang JP, Fan J, Yang LL (2017). Use of 5-fluorouracil loaded micelles and cisplatin in thermosensitive chitosan hydrogel as an efficient therapy against colorectal peritoneal carcinomatosis. Macromol Biosci.

[34] Keese M, Gasimova L, Schwenke K, Yagublu V, Shang E, Faissner R (2009). Doxorubicin and mitoxantrone drug eluting beads for the treatment of experimental peritoneal carcinomatosis in colorectal cancer. Int J Cancer.

[35] Yagublu V, Caliskan N, Lewis AL, Jesenofsky R, Gasimova L, Löhr JM (2013). Treatment of experimental pancreatic cancer by doxorubicin-, mitoxantrone-, and irinotecan-drug eluting beads. Pancreatology.

[36] Tsai M, Lu Z, Wientjes MG, Au JL (2013). Paclitaxel-loaded polymeric microparticles: quantitative relationships between in vitro drug release rate and in vivo pharmacodynamics. J Control Release.

[37] Fan R, Tong A, Li X, Gao X, Mei L, Zhou L (2015). Enhanced antitumor effects by docetaxel/LL37-loaded thermosensitive hydrogel nanoparticles in peritoneal carcinomatosis of colorectal cancer. Int J Nanomed.

[38] Cherukula K, Bae WK, Lee JH, Park IK (2018). Programmed 'triple-mode' anti-tumor therapy: Improving peritoneal retention, tumor penetration and activatable drug release properties for effective inhibition of peritoneal carcinomatosis. Biomaterials.

[39] Kinoshita J, Fushida S, Tsukada T, Oyama K, Watanabe T, Shoji M (2014). Comparative study of the antitumor activity of Nab-paclitaxel and intraperitoneal solvent-based paclitaxel regarding peritoneal metastasis in gastric cancer. Oncol Rep.

[40] Bae WK, Park MS, Lee JH, Hwang JE, Shim HJ, Cho SH (2013). Docetaxel-loaded thermoresponsive conjugated linoleic acid-incorporated poloxamer hydrogel for the suppression of peritoneal metastasis of gastric cancer. Biomaterials.

[41] Han TS, Hur K, Choi B, Lee JY, Byeon SJ, Min J (2017). Improvement of anti-cancer drug efficacy via thermosensitive hydrogel in peritoneal carcinomatosis in gastric cancer. Oncotarget.

[42] Wang Y, Gong C, Yang L, Wu Q, Shi S, Shi H (2010). 5-FU-hydrogel inhibits colorectal peritoneal carcinomatosis and tumor growth in mice. BMC Cancer.

[43] Fan R, Li X, Deng J, Gao X, Zhou L, Zheng Y (2016). Dual drug loaded biodegradable nanofibrous microsphere for improving anti-colon cancer activity. Sci Rep.

[44] Fan R, Wang Y, Han B, Luo Y, Zhou L, Peng X (2014). Docetaxel load biodegradable porous microspheres for the treatment of colorectal peritoneal carcinomatosis. Int J Biol Macromol.

[45] Gunji S, Obama K, Matsui M, Tabata Y, Sakai Y (2013). A novel drug delivery system of intraperitoneal chemotherapy for peritoneal carcinomatosis using gelatin microspheres incorporating cisplatin. Surgery.

[46] Bae WK, Lee JH, Lee SJ, Park MS, Hwang JE, Shim HJ (2011). Enhanced anti-cancer effect of 5-fluorouracil loaded into thermo-responsive conjugated linoleic acid-incorporated poloxamer hydrogel on metastatic colon cancer models. J Nanosci Nanotechnol.

[47] Chen CH, Kuo CY, Chen SH, Mao SH, Chang CY, Shalumon KT (2018). Thermosensitive injectable hydrogel for simultaneous intraperitoneal delivery of doxorubicin and prevention of peritoneal adhesion. Int J Mol Sci.

[48] Xu S, Fan H, Yin L, Zhang J, Dong A, Deng L (2016). Thermosensitive hydrogel system assembled by PTX-loaded copolymer nanoparticles for sustained intraperitoneal chemotherapy of peritoneal carcinomatosis. Eur J Pharm Biopharm.

[49] Qian H, Qian K, Cai J, Yang Y, Zhu L, Liu B (2019). Therapy for gastric cancer with peritoneal metastasis using injectable albumin hydrogel hybridized with paclitaxel-loaded red blood cell membrane nanoparticles. ACS Biomater Sci Eng.

[50] Bajaj G, Yeo Y (2010). Drug delivery systems for intraperitoneal therapy. Pharm Res.

[51] Mohamed F, Marchettini P, Stuart OA, Sugarbaker PH (2003). Pharmacokinetics and tissue distribution of intraperitoneal paclitaxel with different carrier solutions. Cancer Chemother Pharmacol.

[52] Mohamed F, Stuart OA, Sugarbaker PH (2003). Pharmacokinetics and tissue distribution of intraperitoneal docetaxel with different carrier solutions. J Surg Res.

[53] Matsumura Y, Maeda H (1986). A new concept for macromolecular therapeutics in cancer chemotherapy: mechanism of tumoritropic accumulation of proteins and the antitumor agent smancs. Cancer Res.

[54] Bara M, Joffe AR (2014). The methodological quality of animal research in critical care: the public face of science. Ann Intensive Care.

[55] Gupta SK (2019). A study to assess the methodological quality of in vivo animal experiments published in Indian journal of pharmacology: a retrospective, cross-sectional, observational study. Indian J Pharmacol.

[56] Percie du Sert N, Hurst V, Ahluwalia A, Alam S, Avey MT, Baker M (2020). The ARRIVE guidelines 2.0: updated guidelines for reporting animal research. PLoS Biol.

[57] Derrien A, Gouard S, Maurel C, Gaugler M-H, Bruchertseifer F, Morgenstern A (2015). Therapeutic efficacy of alpha-RIT using a 213Bi-anti-hCD138 antibody in a mouse model of ovarian peritoneal carcinomatosis. Front Med.

[58] Lu Z, Tsai M, Wang J, Cole DJ, Wientjes MG, Au JL (2014). Activity of drug-loaded tumor-penetrating microparticles in peritoneal pancreatic tumors. Curr Cancer Drug Targets.

[59] Cristea MC, Frankel P, Synold T, Rivkin S, Lim D, Chung V (2019). A phase I trial of intraperitoneal nab-paclitaxel in the treatment of advanced malignancies primarily confined to the peritoneal cavity. Cancer Chemother Pharmacol.

[60] Cristea MC, Synold TW, Frankel PH, Rivkin SE, Lim D, Chung VM (2015). Pharmacologic advantage (PA) of intraperitoneal (IP) nab-paclitaxel in patients with advanced malignancies primarily confined to the peritoneal cavity. J Clin Oncol.

[61] Kranenburg O, Kvd S, Hingh I (2021). Peritoneal metastases from colorectal cancer: defining and addressing the challenges. Front Oncol.

[62] Christou N, Auger C, Battu S, Lalloué F, Jauberteau-Marchan MO, Hervieu C (2021). Intraperitoneal chemotherapy for peritoneal metastases: technical innovations, preclinical and clinical advances and future perspectives. Biology (Basel)..

[63] Goodman MD, McPartland S, Detelich D, Wasif SM (2015). Chemotherapy for intraperitoneal use: a review of hyperthermic intraperitoneal chemotherapy and early post-operative intraperitoneal chemotherapy. J Gastrointest Oncolo.

[64] de Bree E (2015). Optimal drugs for HIPEC in different tumors. J buon.

[65] Van Oudheusden TR, Grull H, Dankers PY, De Hingh IH (2015). Targeting the peritoneum with novel drug delivery systems in peritoneal carcinomatosis: a review of the literature. Anticancer Res.

[66] ter Riet G, Korevaar DA, Leenaars M, Sterk PJ, Van Noorden CJ, Bouter LM (2012). Publication bias in laboratory animal research: a survey on magnitude, drivers, consequences and potential solutions. PLoS ONE.

[67] Greenland S (2007). Commentary: On ‘Quality in epidemiological research: should we be submitting papers before we have the results and submitting more hypothesis generating research?’. Int J Epidemiol.

[68] Emoto S, Yamaguchi H, Kamei T, Ishigami H, Suhara T, Suzuki Y (2014). Intraperitoneal administration of cisplatin via an in situ cross-linkable hyaluronic acid-based hydrogel for peritoneal dissemination of gastric cancer. Surg Today.

[69] Yamashita K, Tsunoda S, Gunji S, Murakami T, Suzuki T, Tabata Y (2019). Intraperitoneal chemotherapy for peritoneal metastases using sustained release formula of cisplatin-incorporated gelatin hydrogel granules. Surg Today.

[70] Yu J, Lee HJ, Hur K, Kwak MK, Han TS, Kim WH (2012). The antitumor effect of a thermosensitive polymeric hydrogel containing paclitaxel in a peritoneal carcinomatosis model. Invest New Drugs.

[71] Inoue K, Onishi H, Kato Y, Michiura T, Nakai K, Sato M (2004). Comparison of intraperitoneal continuous infusion of floxuridine and bolus administration in a peritoneal gastric cancer xenograft model. Cancer Chemother Pharmacol.

[72] Tang Q, Wang Y, Huang R, You Q, Wang G, Chen Y (2014). Preparation of anti-tumor nanoparticle and its inhibition to peritoneal dissemination of colon cancer. PLoS ONE.

[73] Soma D, Kitayama J, Konno T, Ishihara K, Yamada J, Kamei T (2009). Intraperitoneal administration of paclitaxel solubilized with poly(2-methacryloxyethyl phosphorylcholine-co n-butyl methacrylate) for peritoneal dissemination of gastric cancer. Cancer Sci.

[74] Herrera VL, Colby AH, Tan GA, Moran AM, O'Brien MJ, Colson YL (2016). Evaluation of expansile nanoparticle tumor localization and efficacy in a cancer stem cell-derived model of pancreatic peritoneal carcinomatosis. Nanomedicine (Lond).

[75] Simón-Gracia L, Hunt H, Scodeller PD, Gaitzsch J, Braun GB, Willmore AM (2016). Paclitaxel-loaded polymersomes for enhanced intraperitoneal chemotherapy. Mol Cancer Ther.

[76] Gong C, Yang B, Qian Z, Zhao X, Wu Q, Qi X (2012). Improving intraperitoneal chemotherapeutic effect and preventing postsurgical adhesions simultaneously with biodegradable micelles. Nanomedicine.

[77] Iinuma H, Maruyama K, Okinaga K, Sasaki K, Sekine T, Ishida O (2002). Intracellular targeting therapy of cisplatin-encapsulated transferrin-polyethylene glycol liposome on peritoneal dissemination of gastric cancer. Int J Cancer.

[78] Wang GP, Guan YS, Jin XR, Jiang SS, Lu ZJ, Wu Y (2010). Development of novel 5-fluorouracil carrier erythrocyte with pharmacokinetics and potent antitumor activity in mice bearing malignant ascites. J Gastroenterol Hepatol.

